# Tree-based differential testing using inferential uncertainty for RNA-seq

**DOI:** 10.1101/gr.279981.124

**Published:** 2025-10

**Authors:** Noor Pratap Singh, Euphy Y. Wu, Jason Fan, Michael I. Love, Rob Patro

**Affiliations:** 1 Department of Computer Science, University of Maryland, College Park, Maryland 20742, USA;; 2 Department of Biostatistics, University of North Carolina at Chapel Hill, Chapel Hill, North Carolina 27599, USA;; 3 Department of Genetics, University of North Carolina at Chapel Hill, Chapel Hill, North Carolina 27514, USA

## Abstract

Identifying differentially expressed transcripts poses a crucial yet challenging problem in transcriptomics. Substantial uncertainty is associated with the abundance estimates of certain transcripts which, if ignored, can lead to the exaggeration of false positives and, if included, may lead to reduced power. Here, we introduce a data-driven differential testing method that maximizes biological resolution while retaining statistical power. Given a set of RNA-seq samples, TreeTerminus arranges transcripts in a hierarchical tree structure that encodes different layers of resolution for interpretation of the abundance of transcriptional groups, with uncertainty generally decreasing as one ascends the tree from the leaves. We introduce mehenDi, which utilizes the tree structure from TreeTerminus for differential testing. The nodes output by mehenDi, called the selected nodes, are determined in a data-driven manner to maximize the signal that can be extracted from the data while controlling for the uncertainty associated with estimating the transcript abundances. The identified selected nodes can include transcripts and inner nodes, with no two nodes having an ancestor/descendant relationship. We evaluate our method on both simulated and experimental data sets and compare its performance with other tree-based differential methods, as well as with uncertainty-aware differential transcript/gene expression methods. Our method detects inner nodes that show a strong signal for differential expression, which would have been overlooked when analyzing the transcripts alone.

RNA-seq has become the de facto technology for measuring the expression profiles of different genomic features. Finding the features that differ in expression between the biological samples under different conditions, such as normal versus tumor samples, is called differential analysis and is a fundamental task downstream of quantification. The starting point of differential analysis is to accurately estimate the abundance of the features to be tested, with genes and transcripts being the most common features of interest for most RNA-seq analyses. A gene can express multiple isoforms or transcripts due to alternative splicing, where the transcripts can comprise an overlapping set of exons and thus share sequences. Commonly used RNA-seq quantification methods such as RSEM (Li and Dewey 2011), Salmon (Patro et al. 2017), and kallisto (Bray et al. 2016) probabilistically assign each observed fragment to the transcripts using maximum likelihood or Bayesian inference methods. A probabilistic model is required because a substantial fraction of the sequenced reads can multimap (align similarly or equally well to multiple reference transcripts) as they arise from the regions and sequences shared between the transcripts. Thus, there is ambiguity concerning the true locus of origin of such reads, leading to uncertainty when trying to quantify transcript abundances. Uncertainty can also exist for gene abundance estimates when the reads belong to shared sequences within the genes (e.g., homologous genes); however, in general, gene-level estimates will be more precise compared to transcripts. One approach that estimates the uncertainty associated with the measurement of the abundance estimates is to generate additional samples called inferential replicates through different sampling strategies. These include bootstrap sampling or posterior sampling using MCMC/Gibbs sampling. Such capabilities are provided by several existing quantification methods (Li and Dewey 2011; Turro et al. 2011; Bray et al. 2016; Patro et al. 2017).

The robustness and accuracy of any downstream analysis, such as differential testing, is directly impacted by the quality of the abundance estimates. The methods designed for differential transcript expression testing that utilize inferential replicates report a more robust performance than the methods that do not include them (Seesi et al. 2014; Mandric et al. 2017; Pimentel et al. 2017; Zhu et al. 2019; Baldoni et al. 2024). However, if inferential replicates are incorporated, then we might observe reduced power for the transcripts that exhibit high uncertainty, because we might not be confident of the observed differential signal. In such cases, we might be able to discover more existing differential signals by aggregating transcripts that share reads together into a transcript group. This idea was first proposed in mmcollapse (Turro et al. 2014), also described by Robert and Watson (2015), and further expanded in Terminus (Sarkar et al. 2020), where these groups that contain multiple transcripts, rather than single transcripts, form the feature set for differential analysis.

Expanding upon the above motivation, we recently published TreeTerminus (Singh et al. 2023), which outputs a forest of transcript trees for the samples in an RNA-seq experiment. The leaves represent the transcripts, and the inner nodes represent the aggregated group of the constituent transcripts rooted at each node. The tree encodes different layers of resolution for the interpretation of the abundance of transcriptional groups, with uncertainty generally decreasing as the tree is ascended. This tree can be utilized to find differentially expressed nodes between conditions of interest, where the nodes can consist of both leaves (transcripts) and inner nodes (transcript groups). It is possible that, if the differential signal exists at a transcript, then it might also propagate at the inner nodes. A finer resolution, that is, being as close as possible to the leaves, is, however, preferable for many analyses. The inner nodes should be only part of the output when they constitute the subsets of transcripts whose signal gets masked, primarily due to high uncertainty. Otherwise, the transcripts and not their parent nodes should be selected. Thus, a tree-based differential testing method should be able to find such nodes in a data-driven manner.

Although several tree-based differential testing methods have been proposed, they differ in the error rates they control, the underlying objective functions they seek to optimize, and the data modalities they intend to target. One class of methods focuses on testing for the global null hypothesis, meaning that, for a given inner node, the null hypothesis for all the descendant leaves is true (Miecznikowski and Wang 2023). These methods use a top-down strategy, where they descend an inner node only if the null hypothesis at that node is rejected and continue testing until the null hypothesis cannot be rejected or a leaf node is reached (Meinshausen 2008; Yekutieli 2008; Goeman and Solari 2010; Lynch and Guo 2016; Bogomolov et al. 2021). Whereas some methods (Meinshausen 2008; Goeman and Solari 2010) control the family-wise error rate (FWER) on the rejected set, others (Yekutieli 2008; Lynch and Guo 2016) control the false discovery rate (FDR). More recently, Bogomolov et al. (2021) introduced and controlled a new error rate called selective false discovery rate, which is the expectation of the proportion of falsely discovered nodes (FDP) across each level of a tree. Applying these methods can lead to accepting the null hypothesis at a node in the tree, which can consist of children that contain the differential signal but exhibit a contrasting direction of sign change between the conditions of interest (e.g., two sibling nodes for an inner node that have similar abundance and strong fold change, but with opposite signs, can reduce or nullify the strength of the signal at the parent node). This is very likely to happen when we do the hypothesis testing or compute the *P*-value at each node of the tree individually. It is possible that we may fail to reject the null hypothesis at the root node, even if there is strong evidence of the signal existing at a node situated at a lower height in the tree.

BOUTH (Li et al. 2022) proposes an alternative to the above approaches by defining a new hypothesis test called the modified null hypothesis and controlling an error rate called the false selection rate. It employs a bottom-up procedure and examines whether, for a node, the null hypothesis is true for all the descendant nodes not previously detected (detected implies that the null hypothesis is rejected) at the lower level. It identifies and outputs “driver” nodes such that none of its ancestor nodes is detected. If the driver nodes were to be analyzed, then the features might provide a coarse-grained level of analysis, especially if a clearer signal already exists at a lower height in the tree. On the other hand, it is not clear which descendant nodes corresponding to a driver node should be analyzed to provide a finer resolution of analysis.

Another category of methods aims to leverage the tree structure to enhance power and control the FDR at the leaf level (Xiao et al. 2017; Huang et al. 2021; Bichat et al. 2022). StructFDR (Xiao et al. 2017)) and zazou (Bichat et al. 2022) initially convert the *P*-values into *z*-scores and then propose smoothing on the leaves using a tree-based correlation structure. treeclimbR (Huang et al. 2021) proposes multiple candidate sets, performs multiplicity correction on each set, and selects a final candidate set based on a set of criteria. It rejects the null hypothesis for all the leaves belonging to the inner nodes in the selected candidate set. TEAM (Pura et al. 2023) also proposes another approach for controlling FDR on an aggregated tree but in a different context than the methods described so far. The leaves in the tree are called bins, which consist of cells from both conditions. The tree structure aggregates bins and not the features as we go higher up the tree. The null hypothesis tested at each bin is if the PDF of features is different between the two populations in that bin. If the null hypothesis at a bin cannot be rejected at a lower level, then such bins are aggregated and the hypothesis is tested at the aggregated bin. Importantly, if the aggregated bin is rejected at a higher level in the tree, then the method rejects all the underlying leaf bins. Thus, all these methods cannot be directly applied to our use case. There is uncertainty in measuring the abundance estimate of the transcripts; thus, we do not know which transcript corresponding to the node may be differentially expressed. As a result, passing the information from an inner node to all the descendant leaves might lead to an exaggeration of the false positives or missing the branches where there exists a signal at a node located at a higher height in the tree, to control for the false positives on the leaf set.

There exists another class of methods that fit regression models to compositional data for the covariates using a tree-guided regularization in a maximum likelihood (trac [Bien et al. 2021]) or Bayesian setting (tascCODA [Ostner et al. 2021]). For trac, the parsimony of feature selection (where more nodes at greater heights result in a lesser number of total nodes) depends on the weight assigned to regularization during model fitting. For both trac and tascCODA, when an inner node is selected, its learned coefficients are passed equally to its underlying leaves. In addition to some of the limitations mentioned above that also exist for these methods, both of these methods do not produce *P*-values. This can complicate comparisons with other methods. trac requires cross-validation to determine appropriate hyperparameters for the model, thus requiring a substantial number of samples, which may not be feasible for many RNA-seq experiments. tascCODA also does not scale computationally for data sets with a large number of features, such as RNA-seq.

To overcome some of the above limitations, we introduce our differential testing method, mehenDi, designed to output a set of nodes, called the selected nodes, from the tree. The selected nodes are differentially expressed and can comprise both transcripts and inner nodes, with no two nodes having an ancestor/descendant relationship. It utilizes the tree(s) obtained from TreeTerminus and applies the Swish method for hypothesis testing, accounting for inferential uncertainty (although it is conceptually compatible with other differential testing methods that incorporate inferential uncertainty). The selected nodes are determined in a data-driven fashion by traversing the tree in a top-down manner using a set of rules to maximize the signal that can be extracted from the data while controlling for the uncertainty associated with measuring the abundance estimates. The selected inner node represents a subset of transcripts whose signals are masked at lower levels due to inferential uncertainty; thus, we do not report the differential status of the constituent transcripts.

## Results

### Overview

**Figure 1.**
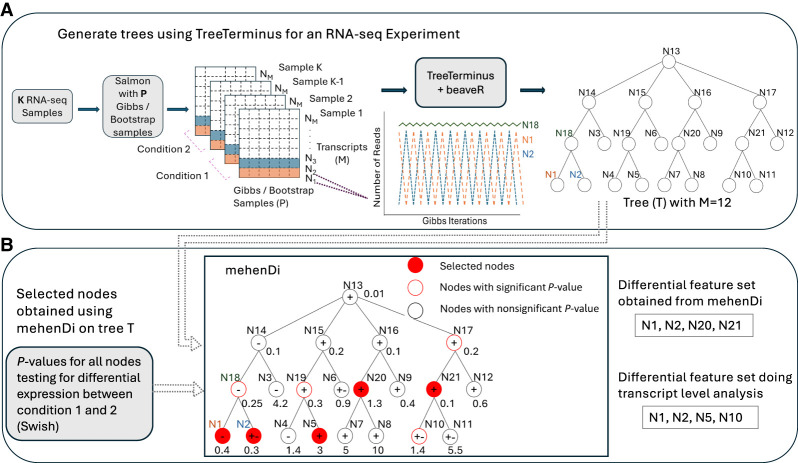
Schematic overview of mehenDi. (*A*) Given a set of RNA-seq samples, TreeTerminus (along with beaveR) creates a tree that encodes the uncertainty structure by using the Gibbs/Bootstrap samples generated by Salmon to compute uncertainty. (*B*) Given a tree, mehenDi outputs a set of selected nodes. The selected nodes are differentially expressed between the conditions of interest and can consist of transcripts and inner nodes, with no two nodes having an ancestor/descendant relationship. Using an example, with a tree having 12 leaves to demonstrate mehenDi, a highlighted node implies that it is selected by mehenDi, with a red outlined node indicating that the node has a significant *P*-value. The + or − on a node indicates the direction of sign change between the conditions, whereas +− indicates we are unsure about the direction. The values plotted along the nodes represent their mean inferential relative variance across the samples (meanInfRV). The meanInfRV threshold in this example is 0.4. The nodes N1 and N2 are chosen instead of node N18 because the meanInfRV of one of its children, node N2, is below the threshold of 0.4. The node N5 is chosen instead of the node N19 because the children have opposite sign changes. Node N20 is the only significant node in its branch and meets all the required criteria. Node N21 is picked because it has at least one nonsignificant child node.

There can exist substantial uncertainty in estimating the abundance of the transcripts due to the presence of reads that can map equally well to shared regions of sequence between transcripts. This quantification uncertainty, in turn, can impact the quality and robustness of differential analysis using transcripts as features. mehenDi provides a different approach to tackle this problem for RNA-seq data. For a given set of samples belonging to an RNA-seq experiment, TreeTerminus (Singh et al. 2023) arranges transcripts in a hierarchical tree structure/s based on quantification uncertainty (Fig. 1A), with the leaves representing the transcripts and an internal node representing an aggregation of the transcripts underlying the node. The uncertainty is computed using the Gibbs/Bootstrap samples (called inferential replicates) which are generated for the RNA-seq samples using Salmon. mehenDi has been designed to perform differential testing directly over the tree(s) produced by TreeTerminus. The uncertainty across the samples generally decreases on ascending the tree(s). Although the procedure for tree construction may result in a forest of trees, a single unified tree is desired for downstream analysis; this is constructed using the R package beaveR (https://github.com/COMBINE-lab/beaveR).

As input, mehenDi requires the *P*-values (computed through an uncertainty-aware differential testing method), the direction of sign change, and mean inferential relative variance (metric to quantify uncertainty) for all nodes in the tree (Fig. 1B). It uses a top-down procedure to output a set of selected nodes that are differentially expressed between the conditions of interest, which seek to maximize the signal that can be extracted from the data while controlling for the uncertainty associated with estimating the transcript abundances. The selected nodes can include transcripts and inner nodes (transcript groups), with no two nodes having an ancestor/descendant relationship. Intuitively, mehenDi allows reporting differential expression at the level of transcript groups (groups of structurally related transcripts) and finds signals that may have been too weak to determine at the level of individual transcripts and which, at the gene level, may involve the aggregation of other structurally dissimilar transcripts not involved in the differential regulation. Figure 1B also provides a toy example demonstrating mehenDi. The exact procedure is described in detail in the Methods section.

### Running mehenDi on null simulation

We recommend computing *P*-values for leaves and inner nodes separately (see Methods) before running mehenDi. Through this analysis, we want to demonstrate why we recommend computing the *P*-values in the specified manner. We also aim to evaluate the false-positive rate for mehenDi specifically comparing it to transcript-level differential analysis.

The simulation framework involves simulating reads from the human transcriptome for 12 samples belonging to two groups (which represent the conditions of interest), with six samples in each group. In the null simulation experiment, the fold change between the groups is fixed at one for all transcripts. We create a total of 10 null simulation experiments. The framework has been described in detail in the Methods section. Thus, by design, the simulation has no true signal for differential expression for the transcripts, and any node deemed as significant by a method would be a false positive.

#### Inner nodes have smaller P-values if the hypotheses testing is carried out on all the nodes of the tree simultaneously

**Figure 2.**
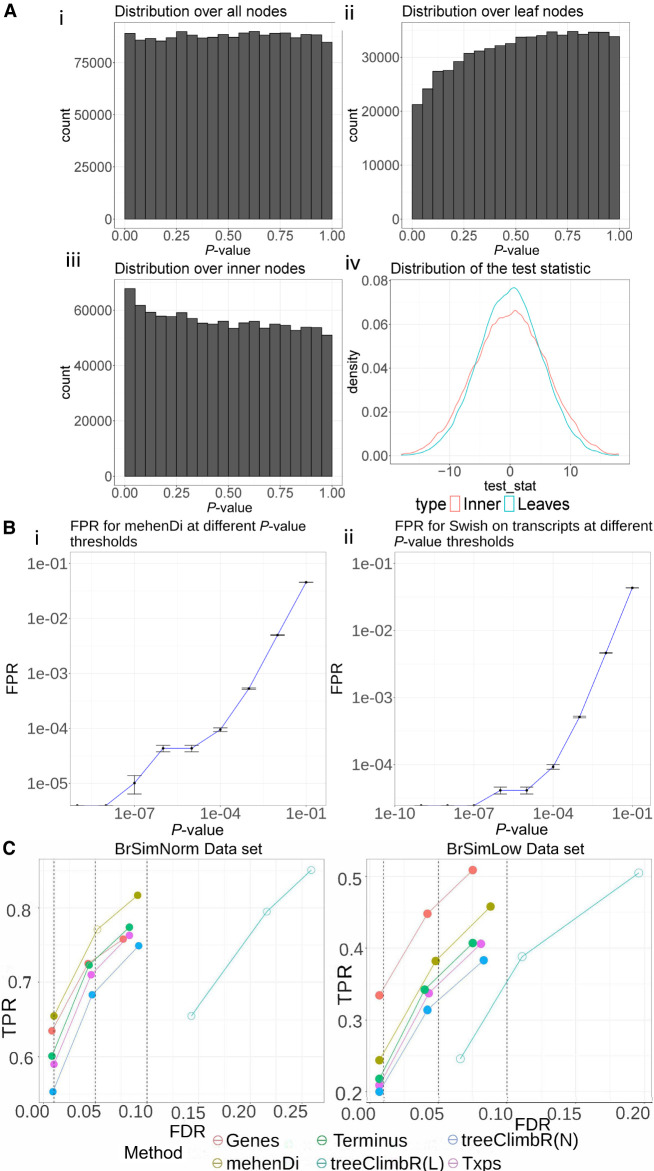
Evaluation on the simulated data sets. (*A*) Distribution of *P*-values and test-statistic for Swish for the null brain simulation. (*i*) Distribution of the *P*-values on the entire node set over the tree when all hypotheses are tested together using Swish. (*ii*) Distribution of the *P*-values over the leaf nodes. (*iii*) Distribution of the *P*-values over the inner nodes. (*iv*) Distribution of the test-statistic on the inner and leaf nodes, which is used by Swish to generate the *P*-values. (*B*) The mean false positive rate (FPR) obtained across the 10 null simulations by varying the *P*-value threshold. The vertical error bar represents the variation around the mean. (*C*) True positive rates and empirical false discovery rates at the different nominal FDR thresholds across the different methods and entities for the BrSimNorm and BrSimLow data sets. Both metrics have been rounded to three decimal places.

For each null simulation, we ran Swish on all the tree nodes simultaneously, and then the computed *P*-values across all the null simulations were concatenated into one vector. As expected, the distribution of the *P*-values across all the nodes is uniform (Fig. 2A, i). However, if we look at the distribution of the *P*-values at the leaves, there is a shift in the distribution towards the right for the leaf *P*-values and the left for the inner node *P*-values (Fig. 2A, ii,iii). Ideally, irrespective of the subset of features that are picked, the distribution should be uniform. This happens because the width of the distribution of the test-statistic which is used by Swish is wider for the inner nodes compared to the leaves (Fig. 2A, iv). Thus, if the *P*-values are generated together for all nodes, we might inflate false positives for the inner nodes and false negatives for the leaves. We thus computed the *P*-values for the leaves and inner nodes separately, and, as expected, both of these show a uniform distribution (Supplemental Fig. S1).

#### The false positive rate (FPR) obtained by mehenDi on the tree is comparable to running Swish on only the transcripts

We next looked at the false positive rate reported by mehenDi on the null simulations and compared it with the FPR obtained by running Swish only on the transcripts. We varied the *P*-value threshold from 1*e* − 9 to 1*e* − 1, increasing the threshold by the power of 10, and then computed the FPR across the 10 simulations, shown in Figure 2B. The empirical FPRs obtained across both settings are comparable to each other. This experiment demonstrates that mehenDi should not exaggerate the false positive rate compared to Swish on transcripts.

### Comparing the methods on simulated data sets with signal

We next benchmarked and analyzed the simulated data sets with differential signals, that is, BrSimNorm and BrSimLow data sets (see Methods), which, again, consist of 12 samples belonging to two groups. Specifically, transcripts in BrSimLow show a relatively weaker signal for differential expression on average compared to BrSimNorm.

#### Features obtained from mehenDi show good sensitivity while controlling for FDR

We compared the features obtained after differential testing using different methods. The features consist of genes (*Genes*), transcripts (*Txps*, treeclimbR[L]), and transcript-groups (*Terminus*, *mehenDi*, treeclimbR[N], *BOUTH*). Txps refers to the transcripts obtained by running vanilla Swish only on the transcripts. When treeclimbR selects an inner node as a candidate node, this implies that all of its descendant transcripts are also differentially expressed. Thus, in our analysis, we compared two feature sets corresponding to treeclimbR: treeclimbR(N), which consists of only the best candidate nodes, and treeclimbR(L), which consists of the underlying transcripts belonging to the nodes. This enabled us to compare the performance of treeclimbR at both the node and transcript levels. On all the simulated and experimental data sets, BOUTH outputs the root node as the driver. Because the root node represents the aggregation of all the transcripts, interpreting the results from BOUTH does not provide any information. We first looked at the number of true-positive transcripts that are covered by the different methods on the simulated data sets (Supplemental Figs. S2, S3) (excluding BOUTH). We observed that mehenDi yields the largest number of true positives, followed by treeclimbR.

We next computed TPR and FDR at different nominal FDR thresholds on the feature sets obtained from the different methods (Fig. 2C). For the BrSimNorm data set, treeclimbR(L) and mehenDi have the largest TPR, whereas for the BrSimLow data set, Genes has the highest TPR, followed by treeclimbR(L) and mehenDi. treeclimbR(L) has an inflated FDR on both data sets (because all transcripts belonging to a candidate node might not be true positives), whereas treeclimbR(N), while controlling for FDR, does not seem to lead to that much overall improvement in sensitivity compared to Txps. mehenDi seems to have a good sensitivity while controlling for the FDR at the same time, with FDR only going slightly beyond the nominal FDR threshold of 0.05 for the BrSimNorm data set. We have excluded the results of BOUTH in the figure because it outputs the root node at all the different nominal FDR values we have tried in our experiments. By design, the root node is not significant in the simulation, and thus the TPR and FDR for BOUTH are 0 and 1, respectively.

We also individually varied the parameters minP and mIrvThresh (see Methods) for mehenDi and observed how the performance changed for the BrSimNorm data set, shown in Supplemental Figure S4. We first kept the parameter *mIrvThresh* constant to its default value 0.4 and varied *minP* from 0.6 to 0.9, incrementing it at an interval of 0.05. We, however, did not observe much change in the performance, with a very slight increase in TPR and FDR as *minP* is increased. We next kept the parameter *minP* constant to its default value 0.7 and varied *mIrvThresh* from 0 to 1, incrementing it at an interval of 0.1. The FDR and TPR both decrease when *mIrvThresh* is increased from 0 to 1, with the FDR for 0.01 controlled after *mIrvThresh* 0.3, for 0.05 after *mIrvThresh* 0.6, and for 0.1 after *mIrvThresh* 0.2. At the lower values of *mIrvThresh*, mehenDi becomes less conservative and prefers nodes with larger heights, which increases the FDR. Higher height nodes are also preferred for larger values of *minP*, because the nodes downstream are more likely not to be assigned a confident sign change. Further, more nodes located at different branches in the tree are also observed for lower values of *mIrvThresh* and higher values of *minP*, compared to selected nodes obtained at the default parameters.

It is important to note that, because the underlying features across the methods are different, these metrics may not be directly comparable. However, we obtained some information about their respective performances. We next investigated the increased sensitivity shown by mehenDi by doing a pairwise comparison with the other methods on the BrSimNorm data set.

#### Features output by mehenDi covers more true-positive transcripts compared to Terminus

**Figure 3.**
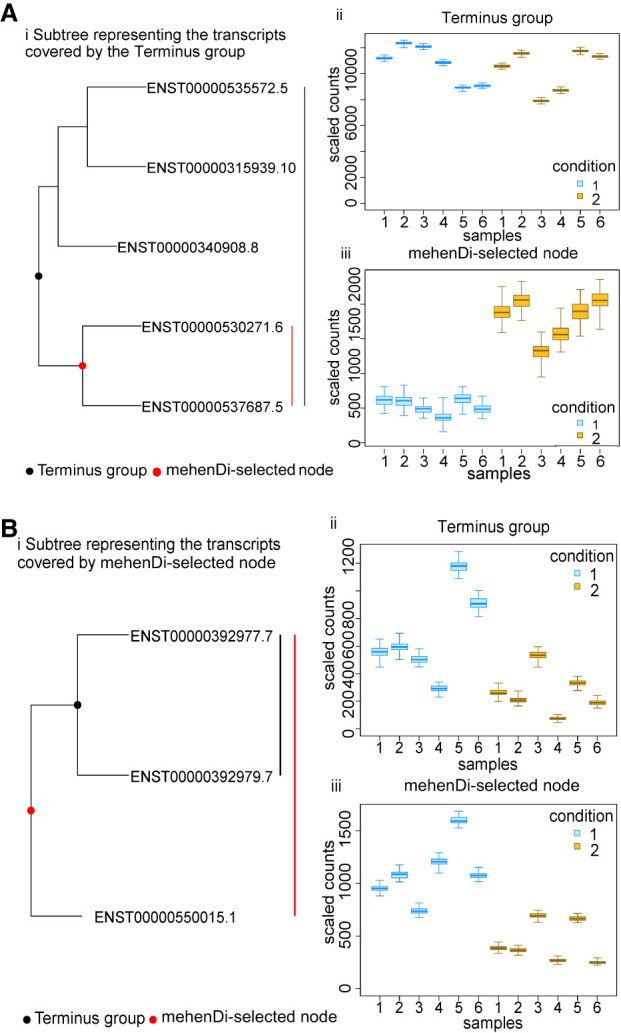
Comparison of mehenDi with Terminus where mehenDi is able to find signal that is lost by Terminus groups. (*A*) Example of a mehenDi node that is overaggregated in Terminus for the BrSimNorm data set. (*i*) Subtree representing the transcripts covered by the Terminus group. (*ii*) Inferential replicates for the Terminus group. (*iii*) Inferential replicates for the selected node output by mehenDi. (*B*) Example of a mehenDi node that is not aggregated enough in Terminus for the BrSimNorm data set. (*i*) Subtree representing the transcripts covered by the mehenDi group. (*ii*) Inferential replicates for the Terminus group. (*iii*) Inferential replicates for the selected node output by mehenDi.

Next, we performed a pairwise comparison between the features output by mehenDi to Terminus obtained at 0.01 nominal FDR. Specifically, we examined the unique true positive transcripts that have been covered by the output features in comparison to one another. We found that features output by Terminus candidate nodes correspond to only 31 unique true-positive transcripts, which map to 26 Terminus groups. For mehenDi, transcripts descending from its selected nodes yield a total of 681 unique true-positive transcripts and correspond to 473 nodes. Although the general expectation might be that most of these additional selected nodes in mehenDi would be as a result of more aggregation, we found 27 nodes, for which the signal exists at a lower level in mehenDi, that get lost in Terminus due to overaggregation. We provide some examples of such cases in Figure 3A and in Supplemental Figures S5 and S6. Similarly, there are 103 such groups (inner nodes in the tree) in mehenDi that contain at least one underlying descendant transcript which was differentially expressed. The differential signal is lost for these transcripts in Terminus, either because the transcripts were not grouped at all or not aggregated enough to produce the signal. In Figure 3B and in Supplemental Figures S7 and S8, we show some examples of such cases.

#### mehenDi finds more unique branches in the tree compared to treeclimbR

We next performed a pairwise comparison between treeclimbR and mehenDi at the 0.01 nominal FDR on the BrSimNorm data set. We first inspected the relative height of the nodes in Supplemental Table S1. We observed more higher-level nodes for treeclimbR compared to mehenDi, with the maximum height of the output node being 19 and 14 in treeclimbR and mehenDi, respectively. It is not trivial to compare these two methods when the nodes output by them are located at different heights and when the differential signal can exist at multiple levels within the same branch in the tree. We thus tried to identify the branches unique to a method using the approach below. For example, imagine that we want to find the nodes unique to mehenDi. To do so, we first extract the nodes that are output by both of these methods (treeclimbR, mehenDi). From the remaining selected nodes (excluding common nodes) that are output by mehenDi, we remove all nodes *i* for which there exists a node *j* that is an output of treeclimbR and is either an ancestor or descendant of node *i* in the tree. The selected nodes that are left represent the unique branches of mehenDi. We repeat a similar process for treeclimbR, to find the unique branches of treeclimbR. We find 199 such nodes unique to mehenDi that map to 258 true positive transcripts, whereas for treeclimbR, we find only two such nodes that map to four true-positive transcripts. Thus, mehenDi finds more unique branches in the tree which, in turn, can enable us to recover the signal from more true-positive transcripts.

#### FDR is not controlled across all the methods on the unique nodes

**Figure 4.**
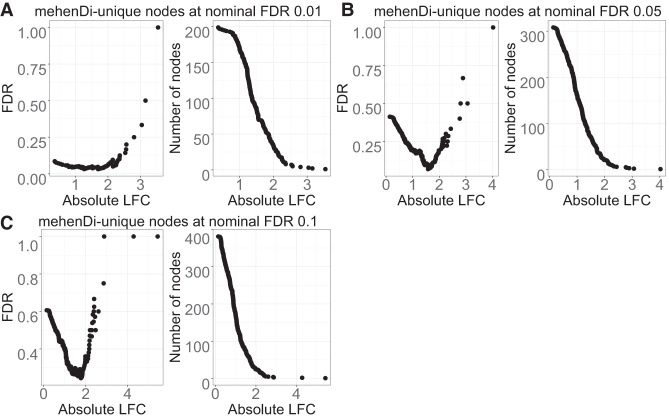
Examination of error metrics for the unique nodes obtained for mehenDi when doing comparison with treeclimbR on the BrSimNorm data set. We vary the magnitude of log fold change (LFC) and plot the empirical FDR and the total number of nodes that are left after filtering the unique nodes based on LFC for the different nominal FDR thresholds. (*A*–*C*) Metrics obtained on nominal FDR 0.01, 0.05, and 0.10.

Because each method can output unique nodes, we next assessed the error rate only on the unique branches/nodes by comparing the methods pairwise. The unique nodes are extracted using the approach described above for the different nominal FDR thresholds. Specifically, we extracted the nodes for pairwise comparison between mehenDi versus treeclimbR, and treeclimbR versus Txps. We provide the observed FDR and the total number of unique nodes for these comparisons in Supplemental Tables S2 and S3. The multiple hypothesis correction methods aim to control the average false discovery proportion of the overall set of rejected hypotheses. However, the error guarantee might not necessarily hold on a specific subset which, in this case, are the unique nodes corresponding to each method. We observed an inflated FDR for the unique features corresponding to Txps and mehenDi, although it decreases for lower nominal FDR thresholds. The observed FDR is also inflated for treeclimbR on pairwise comparison with Txps, containing significantly fewer unique nodes. We next tried to see if we can reduce the FDR by filtering the unique set based on the threshold of absolute log fold change between the conditions of interest. We plotted this in Figure 4 (and in Supplemental Figures S9 and S10), with the panels *A*, *B*, and *C* showing the empirical FDR and the obtained number of nodes for different nominal FDR thresholds. We observed that the observed FDR begins to decrease as the effect size increases, then increases again. The FDR again starts increasing because, after a certain threshold of effect size, only a few nodes are left after filtering. As a result, the false-positive rate increases more rapidly due to a small denominator, leading to a heavier penalty for each incorrect prediction. We thus recommend using a conservative nominal FDR threshold with an appropriate effect size when the novel features unique to any method are analyzed.

### Analyzing the MouseMuscle data set

We next analyzed the MouseMuscle data set which consists of six samples each from EDL and MAST muscle tissues (see Methods). The first two dimensions of the PCA using the top 1000 variable genes for the data set are shown in Supplemental Figure S11.

#### Increase in variation in the parameter values compared to the default setting leads to larger distances between the selected nodes

We first varied the parameters of mehenDi individually and computed the distance of the selected nodes obtained at those parameters from the tree with the selected nodes obtained at the default parameters (Supplemental Fig. S12). For both *minP* and *mIrvThresh*, when the parameter values are closer to the threshold, the distance is lower and increases as the parameters are varied in both directions. The distance increases more sharply when *mIrvThresh* is varied compared to when *minP* is varied. As seen in the BrSimNorm data set, we also observed nodes belonging to distinct branches of the tree and located at higher heights, at low values of *mIrvThresh* and high values of *minP*. The method for computing the distances between the nodes has been described in Supplemental Methods, Section S1.4.

#### mehenDi can find nodes for genes for which the differential signal could not be detected at the transcript level

**Figure 5.**
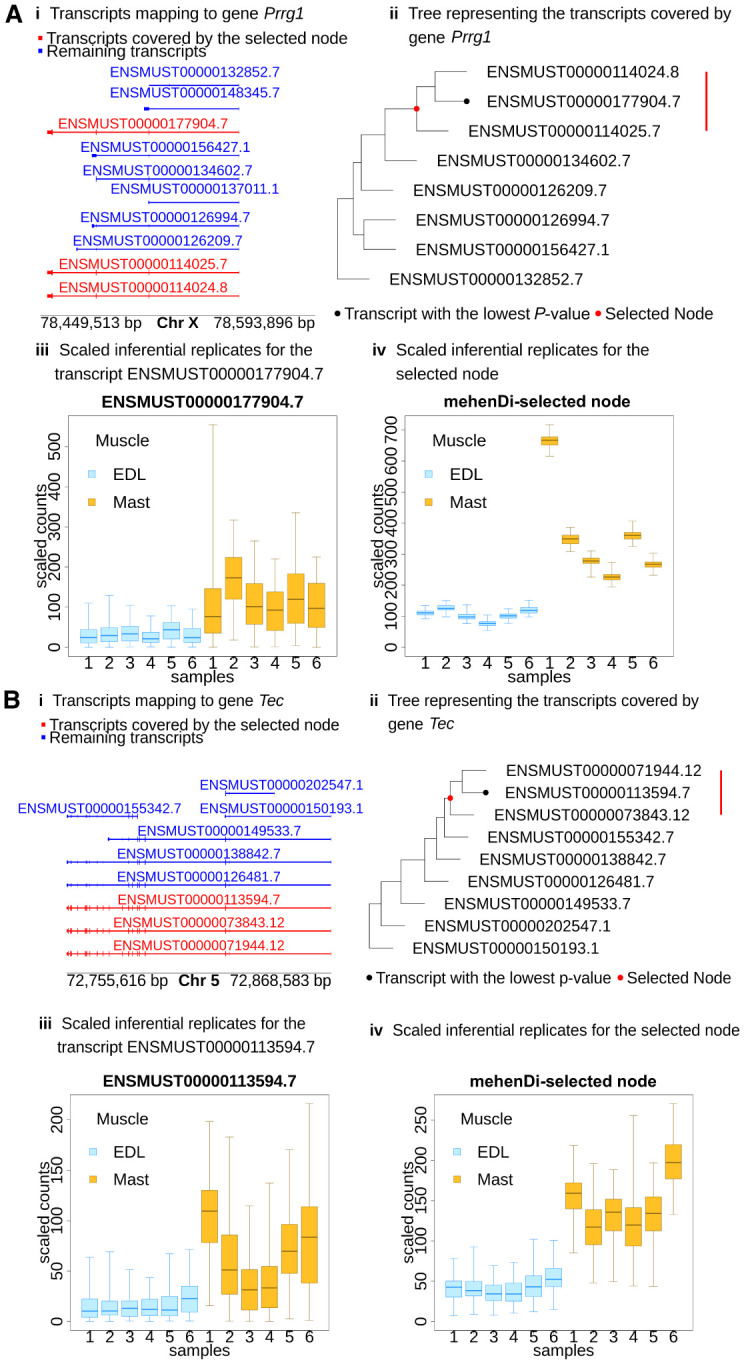
Examining the transcript profile for the gene (*A*) *Prrg1* and (*B*) *Tec* in the MouseMuscle data set. (*i*) Transcripts in a pileup style. (*ii*) Tree representing the transcripts covered by the gene, with the red node representing the transcripts covered by the mehenDi-selected node. (*iii*) Inferential replicates for the transcript which had the lowest *P*-value among all the transcripts in the tree. (*iv*) Inferential replicates for the mehenDi-selected node.

We next compared the nodes obtained by mehenDi at the default parameter values with the different features and the nodes obtained across the other methods. The total number of differentially expressed genes (DEGs), differentially expressed transcripts (DETs) at the different nominal FDRs, and the total number of transcripts that are covered by the nodes/groups output by the different methods at the 0.01 nominal FDR are shown in Supplemental Tables S5 and S6 and Supplemental Figure S13. We observe that mehenDi-reported nodes cover more transcripts compared to treeclimbR-reported nodes. The distribution of the node heights output by mehenDi is shown in Supplemental Table S7, whereas the distribution of the number of unique genes that the transcripts output by the mehenDi map to is provided in Supplemental Table S8 for the different nominal FDR thresholds. We observe that the majority of the output nodes consist of transcripts. Further, most of the transcripts covered by these nodes map to only one gene. We find many genes that are covered by mehenDi nodes which are called significant when differential testing is carried out on the genes, but the underlying transcripts are not called as significant when the testing is performed on the transcripts (Supplemental Table S9). Similarly, when differential testing is carried out on the transcripts, some of the significant transcripts map to nonsignificant genes (Supplemental Table S10). Some of the selected nodes output by mehenDi neither map to a significant gene nor contain a single underlying transcript that is significant (Supplemental Table S11). We also find many selected nodes in mehenDi that map to significant genes, but the genes do not contain a single transcript that is called significant during transcript-level differential analysis (Table 1). We provide some examples for these genes in Figure 5 and Supplemental Figures S14–S18, obtained for 0.01 nominal FDR. For example, we can see that, for the gene *Prrg*1 (Fig. 5A), the transcripts highlighted in red (which underlie the selected node) constitute a very similar set of exons but vary in length. This is reflected in the tree structure in Figure 5A, ii, where the transcripts ENSMUST000000114024.8 and ENSMUST000000177904.7 are aggregated first, followed by transcript ENSMUST000000114025.7. Thus, in Figure 5A, iii, we see that the transcript ENSMUST000000177904.7 has a high inferential relative variance (infRV). This also affects the quality of the signal, which gets stronger at the selected node level constituting the aggregated transcripts (Fig. 5A, iv). We observe a similar aggregation pattern for the gene *Tec* (Fig. 5B), where there is a lot of sequence overlap between the transcripts that underlie the node discovered by mehenDi.

**Table 1.** Number of mehenDi nodes that map to a significant gene, but the gene does not consist of any significant transcripts for the MouseMuscle and the ChimpBrain data set, respectively

	Number of mehenDi nodes	
Nominal FDR	MouseMuscle	ChimpBrain
0.01	309	407
0.05	428	194
0.10	465	213

### Analyzing the ChimpBrain data set

We also assessed the performance of mehenDi on the ChimpBrain data set, which contains five samples each from both the cerebellum and the medial dorsal nucleus of the thalamus (see Methods). The number of genes and transcripts that are differentially expressed between the conditions at the different FDR thresholds is provided in Supplemental Tables S12 and S13 with the total number of features across the different methods shown in Supplemental Figure S19. We again observe that the mehenDi-reported nodes cover the largest number of transcripts. The distribution of the node heights output by mehenDi is shown in Supplemental Table S14, whereas the distribution of the number of unique genes that the transcripts output by mehenDi map to is provided in Supplemental Table S15 for the different nominal FDR thresholds. We observe that the majority of the output nodes consist of transcripts. Further, most of the transcripts covered by these nodes map to only one gene.

#### mehenDi can find nodes for genes for which the differential signal could not be detected at the transcript level

As observed for the MouseMuscle data set, we have many examples for both cases where significantly called transcripts do not necessarily map to significantly called genes and vice versa for significantly called genes (Supplemental Tables S16, S17). Similarly, we find some of the nodes reported by mehenDi map to nonsignificant genes and also do not contain a single transcript that is called significant (Supplemental Table S18).

**Figure 6.**
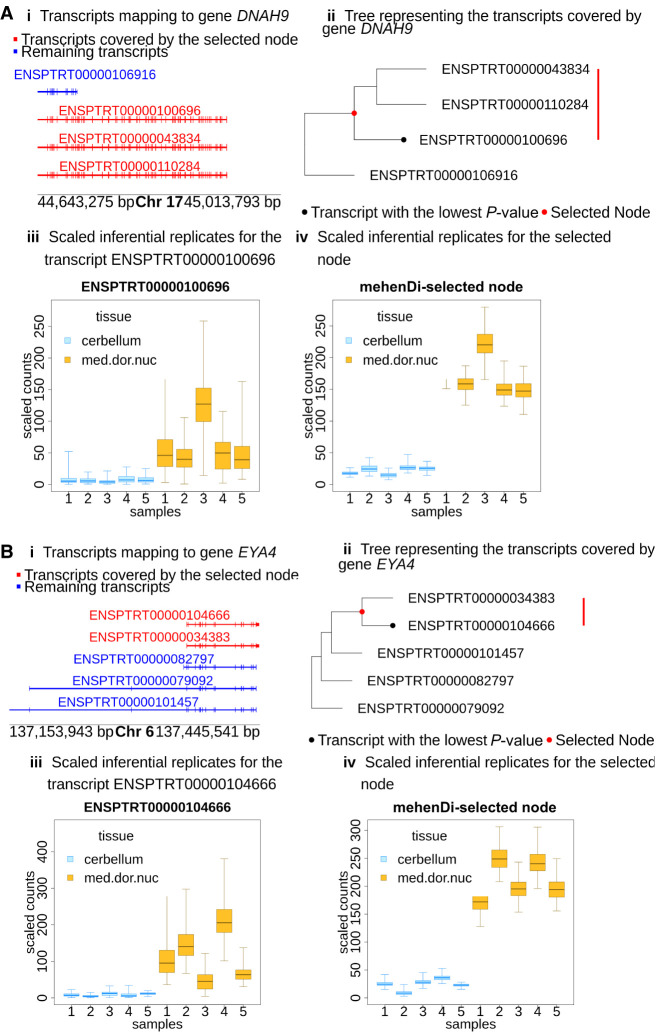
Examining the transcript profile for the gene (*A*) *DNAH9* and (*B*) *EYA4* in the ChimpBrain data set. (*i*) Transcripts in a pileup style. (*ii*) Tree representing the transcripts covered by the gene, with the red node representing the transcripts covered by the mehenDi-selected node. (*iii*) Inferential replicates for the transcript with the lowest *P*-value among all the transcripts in the tree. (*iv*) Inferential replicates for the mehenDi-selected node.

On the other hand, many other mehenDi nodes also map to those significant genes whose underlying transcripts were not called significant (Table 1). We report some of these cases in Figure 6 and Supplemental Figures S20–S25 for the 0.01 nominal FDR. For gene *DNAH9* in Figure 6A and gene *EYA4* in Figure 6B, we observe that for their most significant transcripts, ENSPTRT00000100696 and ENSPTRT00000104666, the differential signal is getting masked owing to high uncertainty, as they share a lot of sequence similarity with the other transcripts. For example, for the gene *EYA4*, the transcripts ENSPTRT00000104666 and ENSPTRT00000034383 differ by only one exon in their underlying sequences. The node output by mehenDi, aggregates such transcripts, leading to lower uncertainty, making the differential signal clearer.

### Comparison of running time

We compared the runtime of treeclimbR and mehenDi on the BrSimNorm and the MouseMuscle data sets. For treeclimbR, two functions, namely getCand and evalCand, have to be called to obtain candidates. We executed the getCand function once and then ran the evalCand function at the different nominal FDR thresholds. mehenDi is parallelizable and supports multiple cores. In our comparison, as shown in Table 2, we presented the runtime for getCand computed once and the runtime for evalCand on each nominal FDR threshold. Additionally, we showcased the time taken by mehenDi when running on a single core and on four cores. Notably, the getCand function takes an order of magnitude more time compared to all the other functions. Furthermore, for mehenDi, we observed an increase in runtime as we raised the FDR threshold. The increase may be attributed to the exploration of more branches, as the threshold becomes less stringent. The parallelized version exhibits a similar pattern. We also observe the benefits of parallelization, achieving a more than threefold increase in speed using four cores compared to using a single core.

**Table 2.** Time taken (in hh:mm:ss) by treeclimbR and mehenDi on the BrSimNorm and MouseMuscle data sets

Method		BrSimNorm (hh:mm:ss)	MouseMuscle (hh:mm:ss)
treeclimbR	getCand	37:06:05	15:05:40
	evalCand (α = 0.01)	00:00:51	00:00:28
	evalCand (α = 0.05)	00:00:50	00:00:29
	evalCand (α = 0.1)	00:00:50	00:00:29
mehenDi (cores = 1)	α = 0.01	00:07:16	00:06:49
	α = 0.05	00:08:49	00:12:14
	α = 0.1	00:09:60	00:15:23
mehenDi (cores = 4)	α = 0.01	00:01:58	00:02:22
	α = 0.05	00:02:25	00:03:55
	α = 0.1	00:02:46	00:04:44

## Discussion

The high level of uncertainty that is observed in the abundances of some transcripts during RNA-seq quantification poses a challenge for downstream analyses, particularly for tasks such as differential testing. Various differential testing methods have been proposed that incorporate uncertainty into testing, and these methods have been applied either to transcripts or genes. This approach can occasionally result in overlooking the signal for the features that are difficult to quantify. Our method, mehenDi, makes use of the tree structure computed by TreeTerminus (Singh et al. 2023) to produce the selected nodes that are significant between conditions. mehenDi provides a data-driven procedure for finding a signal at a resolution between the gene and transcript level for RNA-seq differential analysis, where a selected inner node captures the signal for a set of underlying transcripts that have high uncertainty. Although many tree-based differential testing procedures have been proposed, our method, mehenDi, is among the few tailored toward RNA-seq data that requires specific considerations. Our method requires *P*-values for all the nodes of the tree and thus is flexible in the choice of the differential testing method used, although we have used Swish (Zhu et al. 2019) in this study.

We assessed and evaluated mehenDi on both the simulated and experimental data sets. In the variations of the simulated data sets without true signal, we observed that the FPR obtained by mehenDi was comparable to that generated by doing testing only on the transcripts. Similarly, on both variations of simulated data that included true signals, we observed an increased sensitivity for mehenDi while controlling the FDR at or very near the nominal level. The enhanced sensitivity is not only evident quantitatively through the metrics we report but is also reflected in the discovery of more transcript branches on the tree when conducting pairwise comparisons with other methods. A very high sensitivity, but also a very high FDR, is observed for treeclimbR (Huang et al. 2021), which has been designed to control FDR at the leaf level. Similarly, on the experimental data sets, we observed many significant genes that do not contain a single underlying transcript that is deemed significant when doing transcript-level differential analysis but which contribute to nodes reported by mehenDi. This is a reflection on the ability of mehenDi to find signals that would have been lost when doing transcript-level analysis. Shared exons are commonly observed among these transcripts belonging to the selected nodes, increasing our confidence in the approach's utility. We also observed a clear reduction in uncertainty for the aggregated transcript groups compared to the individual transcripts. This can provide biologists the opportunity to narrow down to a set of transcripts for their analysis that belong to the transcript groups, which would have been lost when doing either transcript- or gene-level analysis.

There exist several areas for improvement of our method and also opportunities for developing new and related methods. The difference in the *P*-value distribution observed for the inner and leaf nodes in the null simulation when the *P*-values for all nodes are calculated together suggests the need for a differential testing method that takes the hierarchy of features into account. It seems that, at least in the different simulations that we have created, we are controlling the FDR. However, because we are using the same data set to create the tree and do differential testing, this technically leads to double-dipping (Kriegeskorte et al. 2009), which can lead to an exaggeration of false positives. This could be one of the reasons why we observe an inflated FDR for treeclimbR.

To mitigate double-dipping, a Poisson/negative binomial sampling of the original data into independent partitions of the same dimensions was introduced in an approach called count splitting (Neufeld et al. 2023a,b). The first partition can be used for latent-variable estimation and the second for inference. Incorporating count splitting into mehenDi will be a promising future direction. The challenge, however, will be how to do this without negatively affecting the power, because mehenDi already appears empirically well-calibrated.

There might also be another source of false positives. When doing a multiple-hypothesis correction on a set of hypotheses, the error rate is controlled over the entire set and not on a subset. Using our filtering strategy, we are outputting a subset of hypotheses that have *P*-value below a certain threshold. Focused BH (Katsevich et al. 2023) provides a way of selecting a *P*-value threshold by running the filter for the different *P*-value thresholds and finding the largest threshold at which the computed conservative estimate of the false discovery proportion for the set is less than the desired error rate. The limitation of this approach is that, apart from being conservative, it also puts constraints on the properties of the filters. Further, Focused BH would be computationally slow in its native form, specifically if filters have to be computed over hundreds or thousands of *P*-value thresholds and each filter takes nontrivial time to run. Adapting this approach for mehenDi can also be explored in the future.

Finally, it would be intriguing to explore the development of a TreeTerminus- and mehenDi-like approach in the context of single-cell RNA-seq analysis, where there are quite distinct but related opportunities for data-driven aggregation to increase the power and specificity of differential analysis. Similarly, there are opportunities to extend this framework to other modalities, such as microRNAs, where RNA sequences can often exhibit significant overlap (Morin et al. 2008). Although differential expression analysis has been the focus of this paper, an important future direction would also be to explore the usage of TreeTerminus trees and mehenDi for differential transcript usage (DTU) analysis.

## Methods

### Preliminaries

For a set of samples in an RNA-seq experiment, TreeTerminus (Singh et al. 2023) outputs a forest of trees that summarizes the abundance uncertainty structure across them. The leaves of the individual trees comprise the set of quantified transcripts, where each internal node represents an aggregation of the set of transcripts belonging to the subtree rooted on it, with no two trees having an overlapping set of transcripts/leaves. The mean uncertainty decreases as we ascend the trees. To enable downstream analysis, the trees in the forest are combined to obtain a single unified tree, T. The uncertainty of a node *n* for a sample *m* (leaf or inner) is estimated by the metric inferential relative variance (infRV) (Zhu et al. 2019) as

(1) $$\mathrm{infR}V_{mn}=\frac{\max\left(\sigma_{mn}^{2}\;-\;\mu_{mn},0\right)}{\mu_{mn}+pc}+d,$$

where $\sigma_{mn}^{2},\mu_{mn}$ are the variance and mean computed over the inferential replicates, *pc* is a pseudocount (with a default value of 5), and *d* is a small global shift (with a default value of 0.01). The inferential replicates refer to the bootstrap or Gibbs samples generated by Salmon. The meanInfRV for a node *n* is defined as the mean of infRV computed across the samples *m*. We describe TreeTerminus in more detail in Supplemental Methods.

### Problem statement

For the given tree T, constructed on the set of *P* transcripts with all nodes including the leaves labeled as N={1,…,N} and *N* ≥ *P*, we want to develop a method that outputs a set of selected nodes C⊂N which are differentially expressed between the conditions of interest. We want to leverage the tree to select nodes that may consist of both transcripts and inner nodes, with the nodes determined in a data-dependent manner to maximize the signal from the data while controlling for uncertainty. At the same time, we do not want to overaggregate, especially if the features with signal exist at a lower level in the tree, because a finer resolution is always preferable. Further, any two nodes in the selected set should not share an ancestor/descendant relationship, that is, for any two nodes *n*_*i*_, *n*_*j*_ in $C,\Lambda (n_{i})\cap \Lambda (n_{j})=\emptyset$, where Λ(*n*_*i*_) represents the set of transcripts that are the descendants of the node *n*_*i*_ in the tree T. This ensures that a set of reads is not counted twice in the selected feature set. Our method, mehenDi, is designed to take the above principles into account. The inputs to mehenDi are the tree T, and the *P*-values for differential testing between the conditions, mean inferential relative variance, and the direction of sign change between conditions for all the nodes *N* belonging to T.

It is important to note that, when mehenDi reports an inner node as selected, we do not imply anything about the differential status of its underlying descendant transcripts. The inner node should be interpreted as a standalone feature, the same way a gene is when doing gene-level analysis, where we are not always trying to comment on the differential status of its transcripts. Not all transcripts of a gene might be differentially expressed. A selected inner node might suggest that the signal existed in at least one underlying transcript but was masked due to uncertainty.

### mehenDi description

We will now outline our method, mehenDi. We first compute the *P*-values, meanInfRV, and direction of sign change for all the nodes in the tree. Swish, which employs inferential replicates, is used to compute the *P*-values for the tree nodes. The counts for an inner node are obtained by aggregating the counts of the underlying transcripts. We recommend computing the *P*-values separately for the inner nodes and leaves. For the transcript group corresponding to a given node, the direction of sign change is examined for each inferential replicate. This is achieved by computing the log fold changes between the biological samples corresponding to that inferential replicate, with each biological sample being associated with a fixed label of interest. If a direction emerges in a certain proportion of inferential replicates beyond a threshold, the node is marked as confident, with that direction determining its sign change. Otherwise, the nodes are labeled as nonconfident and are not assigned a direction of sign change. This is controlled by the parameter minP, which is set to 0.70 by default. Tree nodes with *P*-values below a specified threshold (*pthresh*) are identified and labeled as significant. The set of selected nodes, denoted as C, will be a subset of the significant nodes. The *P*-value threshold that is used for determining the significance of a node is computed by applying a multiple-testing procedure such as Benjamini–Hochberg (BH) (Benjamini and Hochberg 1995) on the leaf nodes that controls the FDR on the leaves at rate α.

mehenDi implements a top-down procedure starting from the root node, which first determines the branches which contain at least one significant node. Proceeding with this approach, it traverses each selected branch and, upon encountering a significant inner node, checks for the following criteria:

1. It has at least one nonsignificant node among its children.
2. All the descendant nodes that are marked confident have the same direction of sign change. This also means that all or a subset of descendant nodes can be nonconfident.
3. All the child nodes have a meanInfRV above a certain threshold (*mIrvThresh*).

If all three criteria are met, that node is selected, and traversal along this branch terminates. Otherwise, this branch is traversed (recursively), checking the above criteria on each newly encountered significant node until either the node is selected or a leaf is reached. If a leaf is significant, it is added to the list of selected nodes. A small, illustrative example demonstrating the above algorithm is provided in Figure 1B.

#### Explanation of the different criteria

The main intuition behind mehenDi is to aggregate transcripts in a data-driven manner to recover the signal that could have been lost due to uncertainty. The first criterion ensures a finer resolution of analysis because, if all children of a significant node are also significant, then the signal already exists at a lower level and aggregation is unnecessary. Owing to uncertainty, the direction of the sign change of a node can be ambiguous, as it might not be consistent across the inferential replicates. We thus assign a particular direction of sign change (positive/up, negative/down) to the nodes between the conditions of interest, only if we are confident. The direction and confidence of sign change of the descendant nodes should also have an impact in determining if a node is selected by mehenDi upstream. The second criterion helps to find a node having a consistent direction of the sign change across its underlying descendant nodes if they can be determined with certainty. This aids in having a clearer and more consistent biological interpretation of the node. The magnitude of effect size can also become smaller at a node if the underlying transcripts have an opposite direction of sign change and we should descend the tree to select nodes. The third criterion also checks for overaggregation because, if we are certain about the abundance estimates for the nodes, we can confidently assess their differential signal and these nodes should not be aggregated. The default value of the parameter *mIrvThresh* is set to 0.40.

#### Computing the P-values

mehenDi can take *P*-values produced by any differential analysis tool as input. However, in this study, we have used Swish (Zhu et al. 2019) for computing the *P*-values because it takes inferential uncertainty into account. The *P*-values for the leaves and inner nodes are computed separately. The reasoning behind this choice is that the leaves have higher uncertainty compared to the inner nodes, especially the ones that appear higher in the tree (the height of a node refers to the maximum distance between the node and its underlying leaves). This leads to a relatively lower shrinkage of the test-statistic towards 0 for the inner nodes and increases the width of the distribution of the test-statistic used by Swish for computing the *P*-values for the inner nodes compared to those of the leaves. As a result, the *P*-values of the inner nodes will be smaller, on average, compared to the leaves, when the *P*-values are computed together. We also recommend controlling for batch effects in the differential testing method if present in diagnostic plots (e.g., PCA).

### Data sets

We evaluated mehenDi on four different data sets, two simulated and two experimental with differential signals, spanning different reference organisms with differential signals. In addition, we also evaluated mehenDi on simulated human data sets with no differential signal.

#### Simulated human data sets

We used the pipeline defined in Love et al. (2018) to create the simulated data sets. *Polyester* (Frazee et al. 2015) was used to generate the FASTQ files corresponding to the paired-end reads. Each simulated data set consists of 12 samples belonging to two groups, with six samples in each group. TPM estimates were extracted from the GTEx V8 frontal cortex data set to simulate samples with realistic ground-truth transcript-level expression. The TPM estimates were then used to create the count matrix (sim.counts.mat), which was provided as an input to *Polyester* to simulate the reads. The reads were simulated with a realistic fragment GC bias. The dispersion of transcript-level counts is drawn from the joint distribution of mean and dispersion values estimated from GEUVADIS samples (Lappalainen et al. 2013). Eighty percent of genes were set to be null genes, with no change in abundance between the two groups. All the transcripts for the next 10% of the genes were set to be differentially expressed, with all the transcripts belonging to a gene having the same fold change. For the remaining 10% of the genes, a single expressed transcript was selected to be differentially expressed. We created two variations of this simulation by varying the range of fold change: in the first variation, we keep the same range as in Love et al. (2018) from 2 to 6; and in the second variation, the range is kept between 1.4 and 2.8. The first variation is referred to as BrSimNorm and the second as BrSimLow. The second simulation is created to measure the change in performance across the methods when the magnitude of effect sizes between the transcripts is decreased.

For the null simulations, we have a very similar setup, with the key difference that the true fold change of all transcripts across conditions is set to 1.

#### Mouse muscle data set

We also analyzed a data set taken from the skeletal muscle study (NCBI Gene Expression Omnibus [https://www.ncbi.nlm.nih.gov/geo/] accession number GSE100505) (Terry et al. 2018). We downloaded RNA-seq data for the six samples belonging to Masseter (MAST) and the six belonging to Extensor digitorum longus (EDL) muscle tissues, with the accession numbers provided in Supplemental Table S4. All the samples belong to the organism *Mus musculus*. This data set is referred to as MouseMuscle.

#### Chimpanzee brain data set

The final data set that has been analyzed in this study is the RNA-seq data from Sousa et al. (2017), with SynapseID syn7067053, collected from five chimpanzees (*Pan troglodyte*). We refer to this data set as ChimpBrain. We specifically analyzed samples obtained from the cerebellum and medial dorsal nucleus of thalamus brain tissues. The batch effects were observed for the specimen ID when visualizing the first two dimensions of the PCA obtained on the normalized counts matrix. We used svaseq (Leek and 2014) on the normalized counts using two surrogate variables to create a fit. The fit was then used to correct batch effects using the removeBatchEffect function in *limma* (Ritchie et al. 2015) for each scaled and log-transformed inferential replicate. The exponential function was then applied to the batch-effect-corrected inferential replicates, converting counts back to the original scale.

### Experimental setup

#### Pipeline to generate trees

For the ChimpBrain data set, only BAM files were available as raw data, which were converted into FASTQ using the bamToFastq command from BEDTools (Quinlan and Hall 2010). The quality control analysis for ChimpBrain and MouseMuscle data sets was done using FastQC (https://www.bioinformatics.babraham.ac.uk/projects/fastqc/) and MultiQC (Ewels et al. 2016). Salmon indexes were built on GENCODE versions v26, vM25, and Pan_tro 3.0 to analyze human, mouse, and chimpanzee data sets (Frankish et al. 2019; Cunningham et al. 2022), respectively. Salmon was used to quantify and generate Gibbs replicates for the RNA-seq samples. For each sample, 100 Gibbs replicates were generated using a thinning factor of 100. The trees from TreeTerminus were created using the Consensus mode. All the pipelines used for analysis in this paper were constructed using Snakemake (Mölder et al. 2021).

#### Differential expression analysis

We compared the performance of carrying out the differential expression analysis using the different feature sets and tree-based methods on the analyzed data sets. The compared features comprise the selected transcripts (*Txps*), genes (*Genes*), Terminus groups, and the best candidate, driver, and selected nodes obtained by running the different tree-based methods on the unified tree T from TreeTerminus. The base set for Terminus comprises the discrete transcript groups and any remaining transcripts that belong to the transcript set of interest but that are not covered by Terminus groups. For the tree-based methods, we compare mehenDi, treeclimbR, and BOUTH. Median-ratio scaling (Anders and Huber 2010) was used to normalize the counts. When scaling the counts for the nodes in the tree, we first computed the size factor only using the leaf nodes and then divided the bias-corrected scaled counts of the inner nodes by the size factor. This procedure is described in detail in Supplemental Methods, Section S1.3. Swish was then used for generating *P*-values for the normalized counts. Whereas treeclimbR and mehenDi require *P*-values for all the nodes in the tree, BOUTH requires the *P*-values only for the leaf nodes. The *P*-values were generated separately for the leaf and inner nodes using Swish. For treeclimbR, the parameter α was set to the nominal threshold, which aimed to control the FDR at leaf nodes to extract the best candidates. We considered two feature sets for treeclimbR in our evaluation. treeclimbR(N) refers to the best candidate set obtained after running treeclimbR, and treeclimbR(L) consists of the descendant transcripts for the nodes in treeclimbR(N). We also considered treeclimbR(L) in our analysis because treeclimbR is designed to control FDR at the leaf level, which, in this case, corresponds to the transcripts.

For BOUTH, we set the parameter *FAR* to the nominal FDR, which controls for a false assignment rate, and we then extracted the driver nodes from its output. A driver node provides the highest level of resolution with none of their ancestors being associated for differential analysis. Many of the descendant nodes of a driver node at different resolutions can be labeled as detected for differential analysis, which can complicate the analysis when trying to decide which resolution node to select; thus, a driver node was chosen for our analysis. For mehenDi, the nominal FDR threshold was used to select the *P*-value threshold (on the leaves) to obtain the selected nodes. For the other features (*Txps*, *Genes*, *Terminus*), Swish was applied directly to their base sets individually, and the selected set consisted of features that have their adjusted *P*-values less than the given nominal FDR threshold.

#### Evaluation on the simulated data sets

Because the ground truth is known for the simulated data sets, we computed the true positive rate (TPR) and false discovery rate on the selected features obtained from the different methods at the different nominal thresholds (0.01, 0.05, 0.1). When computing these metrics for the features that consist of transcript groups or the inner nodes in the tree, we only considered the status of the differential expression for that node or the transcript group in our evaluation. We did not evaluate the differential status at the underlying descendant transcripts or other inner nodes corresponding to the group or inner node. To obtain the ground truth for differential expression at an inner node, we first created the aggregated counts matrix for the tree T, sim.tree.counts.mat using sim.counts.mat. The sim.counts.mat consists of the true transcript counts corresponding to sample groups, on which we were evaluating the differential expression. This is the same matrix that was provided as an input to *Polyester* to simulate reads. The count of an inner node in sim.tree.counts.mat is computed by aggregating the counts of all the descendant transcripts in sim.counts.mat. The inner node is differentially expressed if the absolute log fold change (LFC) for that node computed using sim.tree.counts.mat is larger than a threshold. The threshold is set to the LFC obtained at the tree's root node in the sim.tree.counts.mat.

### Software availablity

mehenDi is available as an R package (R Core Team 2023) and can be obtained from GitHub (https://github.com/NPSDC/mehenDi) and Zenodo (https://doi.org/10.5281/zenodo.15797161). The scripts to reproduce the results in this work, and to obtain and generate the datasets that have been analyzed, are available at Zenodo (https://doi.org/10.5281/zenodo.11481255 and https://doi.org/10.5281/zenodo.11481348, respectively). All scripts are also available as Supplemental Code.

## Supplemental Material

Supplement 1

Supplement 2

Supplement 3
