# Supplementary material for: Tree-based differential testing using inferential uncertainty for RNA-seq
Source: Genome Res. 2025 Oct;35(10):2326–38. doi: 10.1101/gr.279981.124 (PMC12487820; doi:10.1101/gr.279981.124)

Supplementary File

Tree-based differential testing using inferential uncertainty for RNA-Seq

Noor Pratap Singh[1], Euphy Wu[2], Jason Fan[1], Michael.I Love[2,3], and Rob Patro[1]

npsingh@cs.umd.edu, euphyw@live.unc.edu, jasonfan@cs.umd.edu, michaelisaiahlove@gmail.com, rob@cs.umd.edu

[1]*Department of Computer Science, University of Maryland, College Park*

[2]*Department of Biostatistics, University of North Carolina-Chapel Hill*

[3]*Department of Genetics, University of North Carolina-Chapel Hill*

[*]*Corresponding author: Rob Patro, rob@cs.umd.edu*

S1 Methods

S1.1 Range-factorized equivalence class

An equivalence class denotes an association from a set of transcripts to a set of reads, that are mapped to all the transcripts in that set. A `range-factorized` equivalence class in addition also encodes the mapping quality, with a single class constituting a set of pairs (t_i, w_i) rather than just a set of t_i, where t_i denotes the transcript and w_i represents the average conditional probability with which the fragments in the equivalence class arose from that transcript.

S1.2 TreeTerminus

For a given RNA-Seq experiment consisting of M samples, `TreeTerminus` [6] outputs a forest of K trees $\mathcal{T} = \{T_1, T_2, ..., T_K\}$, that summarize the abundance uncertainty structure across all the M samples. The leaves of the individual trees comprise the set of quantified transcripts and each internal node represents an aggregation of the set of transcripts belonging to the subtree rooted at it, with no two trees having an overlapping set of transcripts/leaves. The input to `TreeTerminus` is the `salmon` [7] quantification estimates, L inferential replicates, and range-factorized equivalence classes [5] corresponding to each RNA-Seq sample m, $m \in \{1, ..., M\}$. The L inferential replicates are produced either through Gibbs sampling or bootstrap sampling and are denoted by $\mathcal{I}_{mi} = \{I_{mi_1}, I_{mi_2}, ..., I_{mi_L}\}$, where I_{mi_l} represents the counts of the transcript i

for sample m at the lth Gibbs/bootstrap iteration. The inferential replicate counts for an inner node are found by adding inferential replicate counts of each constituent transcript individually. The tree represents the order in which the different transcripts are aggregated into transcript groups starting from the leaf nodes and encoding different resolution layers for interpretation of the abundance of transcriptional groups, with uncertainty generally decreasing as one ascends the tree from the leaves. The uncertainty for any node (leaf or inner node) n for a given sample m is estimated using the metric `infRV` defined in [3], over $\mathcal{I}_{mn}$ as:

$$\text{infRV}_{mn} = \frac{\max(\sigma^2_{I_{mn}} - \mu_{I_{mn}}, 0)}{\mu_{I_{mn}} + pc} + d \tag{1}$$

where $\mu_{I_{mn}}, \sigma_{I_{mn}}$ are the mean, variance over the L inferential replicates for a sample m and node n, pc is a pseudocount (with a default value of 5) and d is a small global shift (with a default value of 0.01). The nodes situated at the lower heights in a branch in the tree usually represent the set of nodes for which large reduction in `infRV` was observed compared to its underlying children nodes. For most nodes in the tree, the underlying transcripts belong to the same gene, due to large sequence overlap between them which is a driving factor behind uncertainty. However, the transcripts in a node can also map to different genes, as there can be overlapping sequence regions between different genes as well, and also sequence similarity between genes belonging to a gene family. While a reduction in `infRV` governs how transcripts are aggregated into nodes, it does not mean that all the underlying transcripts will have similar strength and direction of differential signal between the conditions of interest.

Unified Tree - Once the forest from `TreeTerminus` is obtained, a unified tree is constructed. For the sake of simplicity, let $\mathcal{T}$ denote the unified tree. The unified tree is constructed using the `R` package `beaveR`(https://github.com/NPSDC/beaveR). This tree is constructed by first creating a new root node and assigning all trees in the forest and the remaining transcripts in the transcriptome not covered by the trees as children of this root node.

S1.3 Median-ratio scaled counts for the nodes in the tree

Using the formulation from [3], let Y^0 denote the counts matrix obtained for Salmon for the transcript set containing M samples $1, .., m$ and P transcripts $1, .., p$, with Y^0_{ji} representing the counts for transcript j in sample i. Let the matrix $Y^{\mathcal{T}0}$ denote the counts obtained for all the nodes in the tree $\mathcal{T}$ that has P leaf nodes, where for an internal node n, $Y^{\mathcal{T}0}_{ni} = \sum_{d=1}^{|\Lambda(n)|} Y^0_{t_d i}, \forall t_d \in \Lambda(n)$, where $\Lambda(n)$ denote the indexes of the descendant transcripts of node n. The counts $Y^{\mathcal{T}}_{ji}$ are divided by a bias length term b_{ji}, accounting for the length w.r.t other transcripts:

$$b_{ji} = \frac{l_{ji}}{(\prod_{i=1}^{m} l_{ji})^{\frac{1}{m}}}$$

Then we divide the counts by b_{ji} as

$$Y_{ji}^{\mathcal{T}*} = \frac{Y_{ji}^{\mathcal{T}0}}{b_{ji}}$$

The counts are then scaled to the geometric mean of sequencing depth as

$$Y_{ji}^{\mathcal{T}**} = \frac{Y_{ji}^{\mathcal{T}*}}{\sum_{j=1}^{P} Y_{ji}^{\mathcal{T}*}} \times \left(\prod_{i=1}^{m} \sum_{j=1}^{P} Y_{ji}^{\mathcal{T}0} \right)^{\frac{1}{m}}$$

For each sample i, a median-ratio size factor is computed as

$$s_i = \text{median}_j^P \frac{Y_{ji}^{\mathcal{T}**}}{\left(\prod_{k=1}^{m} Y_{jk}^{\mathcal{T}**} \right)^{\frac{1}{m}}}$$

We compute the size factor over only the leaf nodes. The final normalized counts are then computed as:

$$Y_{ji}^{\mathcal{T}} = \frac{Y_{ji}^{\mathcal{T}**}}{s_i}$$

The lengths of the inner nodes are computed using the strategy employed by `summarizeToGene` function in the `R` package `tximport` [4]. The length of an inner node n for sample i is computed as:

$$l_{ni}^{\mathcal{T}} = \frac{\sum\limits_{d=1}^{|\Lambda(n)|} l_{t_d i} tpm_{t_d i}}{\sum\limits_{d=1}^{|\Lambda(n)|} tpm_{t_d i}}, \forall t_d \in \Lambda(n)$$

Here *tpm* refers to transcripts per million estimates that are provided by Salmon.

S1.4 Distance between nodes

The distance between the set of nodes N_d and N_p is computed as :

$$
\begin{aligned}
\mathcal{D}(N_d, N_p) &= \frac{dist(N_d, N_p) + dist(N_p, N_d)}{2}, \\
dist(N_a, N_b) &= \frac{\sum_{i=1}^{|N_a|} d(N_{ai})}{\|N_a\|}, \\
d(N_{ai}) &= \begin{cases} 0 & \text{if } N_{ai} \in N_b \\ Path\_length(N_{ai}, N_{bk}) & \text{if } N_{bk} \in N_b \text{ and } N_{bk} \text{ is either an ancestor} \\ & \text{ or descendent of } N_{ai} \\ Path\_length(N_{ai}, \text{root}) + 1 & \text{if } N_{ai} \notin N_b \text{ and no ancestor or} \\ & \text{descendant of } N_{ai} \text{ exists in } N_b \end{cases}
\end{aligned}
$$

where N_d, N_p denote the node set output by `mehenDi` at the default parameters and parameter set p respectively. $Path\_length(N_{ai}, N_{bk})$ denote the length of the path between the nodes N_{ai} and N_{bk}. We are computing the average distance per node between the two sets. If the same node is present in both sets, the distance between them would be 0. Similarly, if for a node belonging to one set, there exists a node in the other set which is an ancestor/descendant for it, then the distance is computed by calculating the length of the path between them on the tree. On the other hand, if there is no ancestor/descendant for a node in the other set, then the distance is the length of the path from the root to that node with 1 added. 1 is added since this would be the lowest height node in the other set. The nodes that don't have an ancestor or descendant in the other set, can be the largest contributing factor to the distance metric and can create asymmetry for the overall distance metric aka $dist(N_a, N_b) \neq dist(N_b, N_a)$, as they do not directly have a counterpart in the other set. This can skew the metric, depending on the set w.r.t which distance is computed, especially if that set consists of nodes that represent unique branches in the tree. To balance this, our final distance metric $\mathcal{D}(N_d, N_p)$ is the average of $dist(N_d, N_p), dist(N_p, N_d)$.

S2 Note

S2.1 Comparing biological and inferential relative variance and its incorporation by Swish

There exists biological variance for a gene across samples in an experiment, with overdispersion modelled using a negative binomial/quasi-Poisson distribution in many parametric differential testing methods[2], [1]. However, the inferential replicates used by TreeTerminus and mehenDi are generated for each biological sample. These inferential replicates provide a measure of how certain we are about the abundance estimates for a biological sample using the metric of inferential

relative variance (infRV). The inferential replicates mirror the technical replicates, for which the Poisson distribution is assumed (mean same as variance); any deviation from this trend is captured by infRV, with a higher value of this metric denoting a high uncertainty associated with the inference of that transcript's abundance. The infRV is computed for each node (which can be a gene, a transcript, or a transcript-group) for a given biological sample using the inferential replicates. This metric enables our method to create tree(s) for each biological sample individually, such that uncertainty decreases upon ascending the tree(s) or across all biological samples in an RNA-seq experiment. Note that inferential variance for a sample can be high or low regardless of the biological variability seen in the experiment. Swish tries to takes into account both types of variance in its inference procedure: the biological variance is involved when computing the Wilcoxon statistic (for the two group mode) while inferential variance per sample is taken into account when integrating the base test statistic over inferential replicates.

S2.2 Role of equivalence classes in capturing inferential uncertainty

The equivalence class is data-dependent and represents a relationship between the set of reads and transcripts, where each equivalence class contains the reads that map to the same set of transcripts. Thus, equivalence classes are a function of the data and mapping/alignment algorithm, and we do not have a direct choice in the presented method over the equivalence relation that is defined and the classes that are produced. That is, the equivalence classes are a deterministic function of the input data and the alignment algorithm applied. The equivalence classes encapsulate all read-to-transcript mapping uncertainty, conditional on the mapping/alignment method.

Ideally, if each read is mapped uniquely to only a single transcript, then there will be no uncertainty, and the number of equivalence classes will be equal to the total number of unique transcripts that are mapped by the entire read set. The only way an equivalence class may be wrongly formed is if the reads are mapped incorrectly, which is a function of the mapping algorithm. In this case, any quantification algorithm and the corresponding downstream analysis will suffer.

It is possible that inferential replicates might not be able to fully capture the uncertainty profile for certain transcripts due to the limitations of a particular posterior/bootstrap sampling algorithm, but they still provide much more information than a point estimate.

S2.3 Effect of batch effects in capturing uncertainty and on mehenDi output

There can only be two potential places where batch effects might pose an issue, namely, tree construction and downstream differential testing. The batch effects can alter the counts. When it comes to tree construction, the trees are constructed using the metric inferential relative variance, which is computed for each sample separately, thus minimizing the issues which occur due to batch effects, which occur when the counts across samples are compared. Note that the InfRV by construction is stabilized across the mean count. While subtle differences in uncertainty structure might be observed for a given sample due to batch effects, we do not anticipate nor have we ever observed substantial changes in the final tree

structures that are obtained across samples. For the differential analysis, we don't think batch effects would pose an issue that would be unique to our analysis. We can use mehenDi in combination with a testing procedure that controls for batch using standard methods (Swish can also be run stratified across batch, or inferred batch variation can be regressed out of inferential replicate count matrices). We recommend to control for batch effects in the differential testing method if it present in the diagnostic plots (e.g. PCA). Moreover, we provide an explicit example of such in the current manuscript. Specifically, in this manuscript, we have analyzed the ChimpBrain dataset which shows evidence for batch effects.We use the p-values computed by Swish accounting for batch-effects.

References

[1] Mark D Robinson, Davis J McCarthy, Gordon K Smyth. edgeR: a Bioconductor package for differential expression analysis of digital gene expression data. *Bioinformatics* 26(1):139–140, 2010.

[2] Michael I Love, Wolfgang Huber, Simon Anders. Moderated estimation of fold change and dispersion for RNA-seq data with DESeq2. *Genome biology* 15:1–21, 2014.

[3] Anqi Zhu, Avi Srivastava, Joseph G Ibrahim, Rob Patro, Michael I Love. Nonparametric expression analysis using inferential replicate counts. *Nucleic Acids Research* 47(18):e105–e105, 2019.

[4] Charlotte Soneson, Michael I Love, Mark D Robinson. Differential analyses for RNA-seq: transcript-level estimates improve gene-level inferences. *F1000Research* 4, 2015.

[5] Mohsen Zakeri, Avi Srivastava, Fatemeh Almodaresi, Rob Patro. TreeTerminus—creating transcript trees using inferential replicate counts. *Bioinformatics* 33(14):i142–i151, 2016.

[6] Noor Pratap Singh, Michael I Love, Rob Patro. TreeTerminus—creating transcript trees using inferential replicate counts. *iScience* 26(6), 2023.

[7] Rob Patro, Geet Duggal, Michael I Love, Rafael A Irizarry, Carl Kingsford. Salmon provides fast and bias-aware quantification of transcript expression. *Nature methods* 14(4):417–419, 2017.

S3 Figures

Figure S1: Distribution of the p-values for the leaf and inner nodes on the null simulations, when the hypothesis testing is carried out separately.

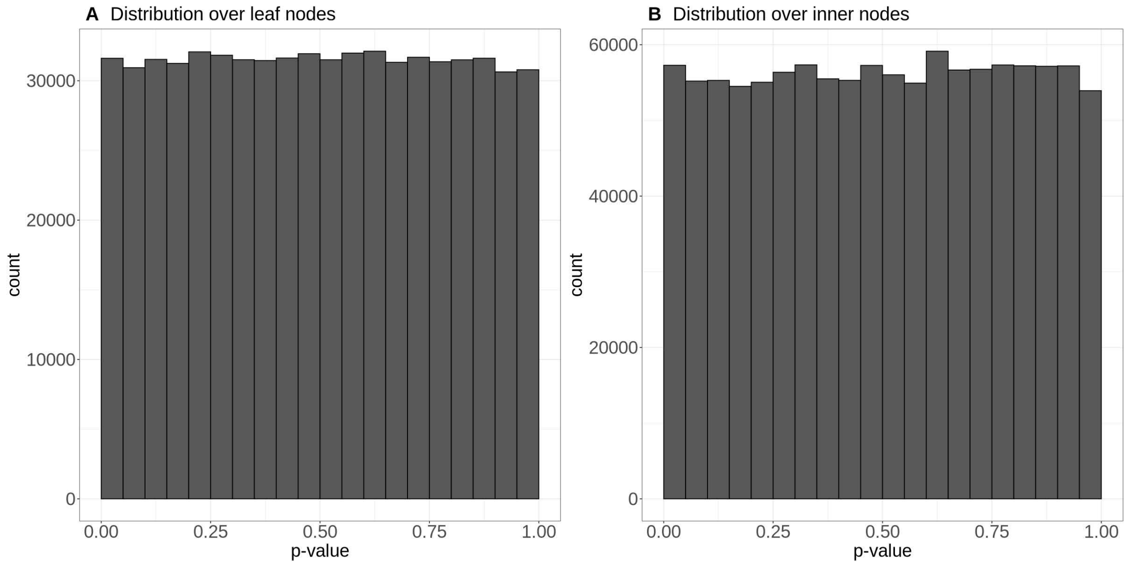

Figure S2: UpSet plot covering the number of true positive transcripts that are covered by the nodes that are output by the different methods for the `BrSimNorm` dataset nominal FDR.

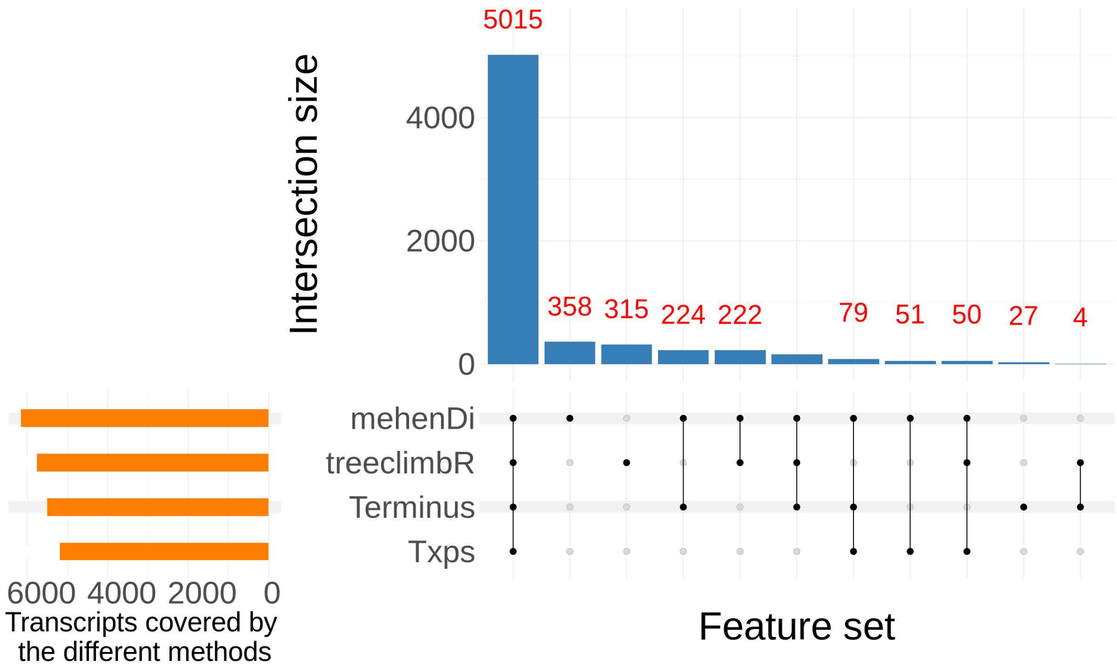

Figure S3: UpSet plot covering the number of true positive transcripts that are covered by the nodes that are output by the different methods for the `BrSimLow` dataset at the 0.01 nominal FDR.

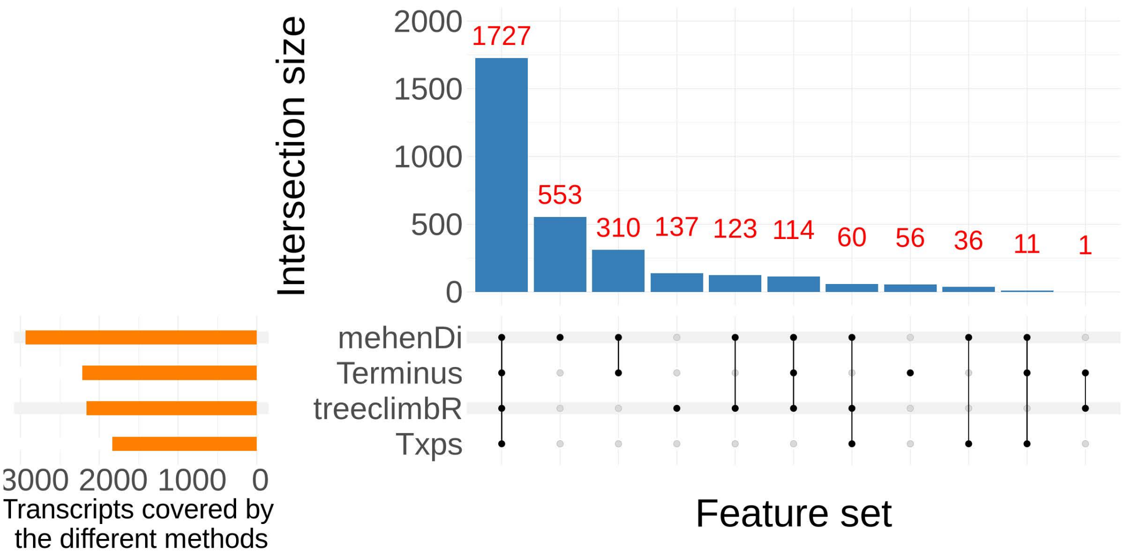

Figure S4: True Positive Rates and Empirical False Discovery Rates at the different nominal FDR thresholds by individually varying the parameters `minP` and `mIrvThresh` for the `BrSimNorm` dataset. Both the metrics have been rounded to 3 decimal places

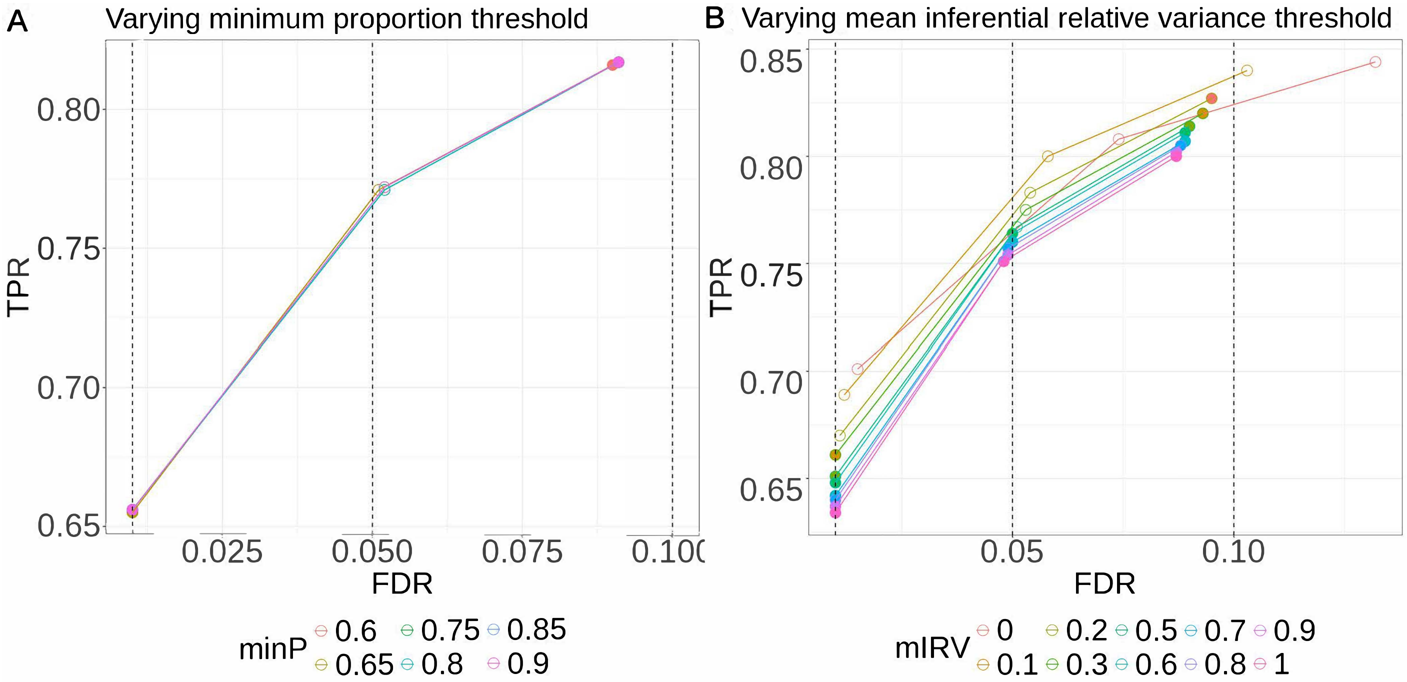

Figure S5: Example of a `mehenDi` node that is overaggregated in `Terminus` for the `BrSimNorm` dataset. (A) Subtree representing the transcripts covered by the `Terminus` group. (B) Inferential replicates for the `Terminus` group. (C) Inferential replicates for the selected node output by `mehenDi`.

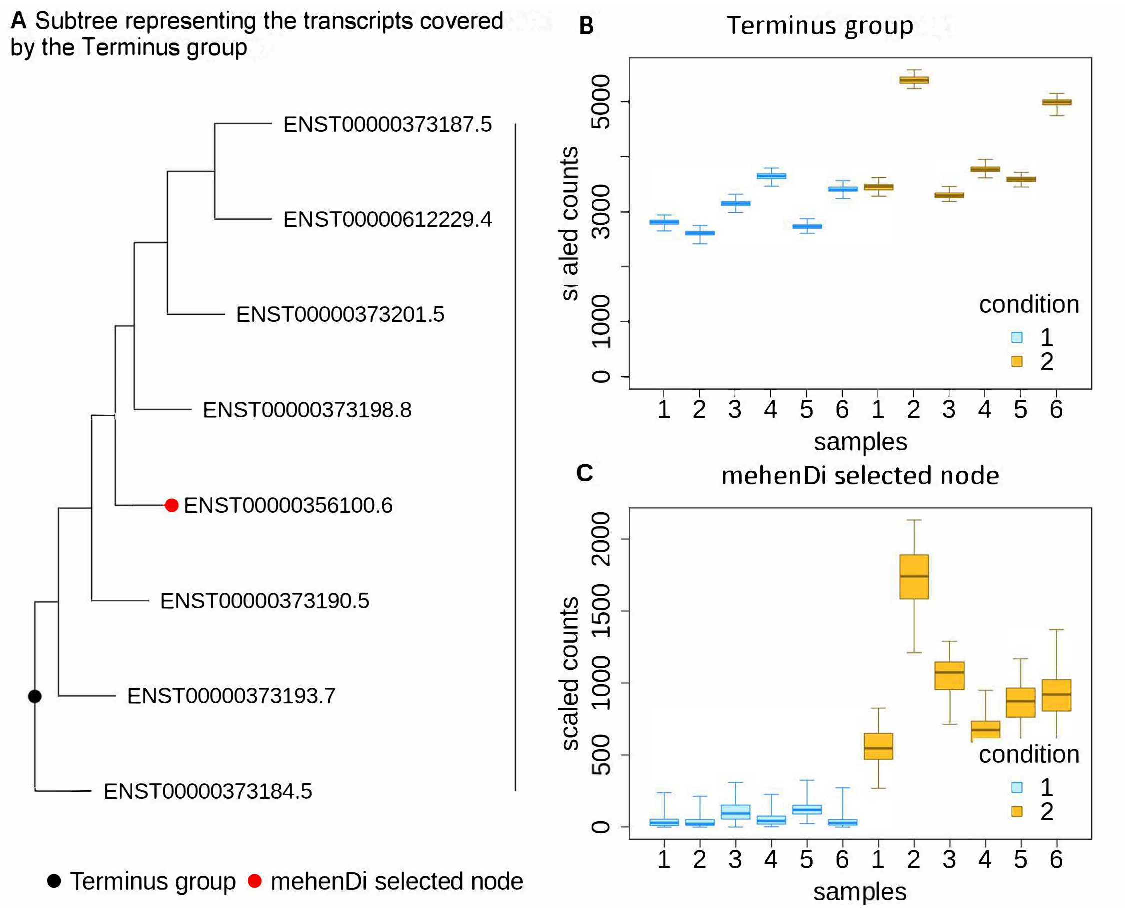

Figure S6: Example of a `mehenDi` node that is overaggregated in `Terminus` for the `BrSimNorm` dataset. (A) Subtree representing the transcripts covered by the `Terminus` group. (B) Inferential replicates for the `Terminus` group. (C) Inferential replicates for the selected node output by `mehenDi`.

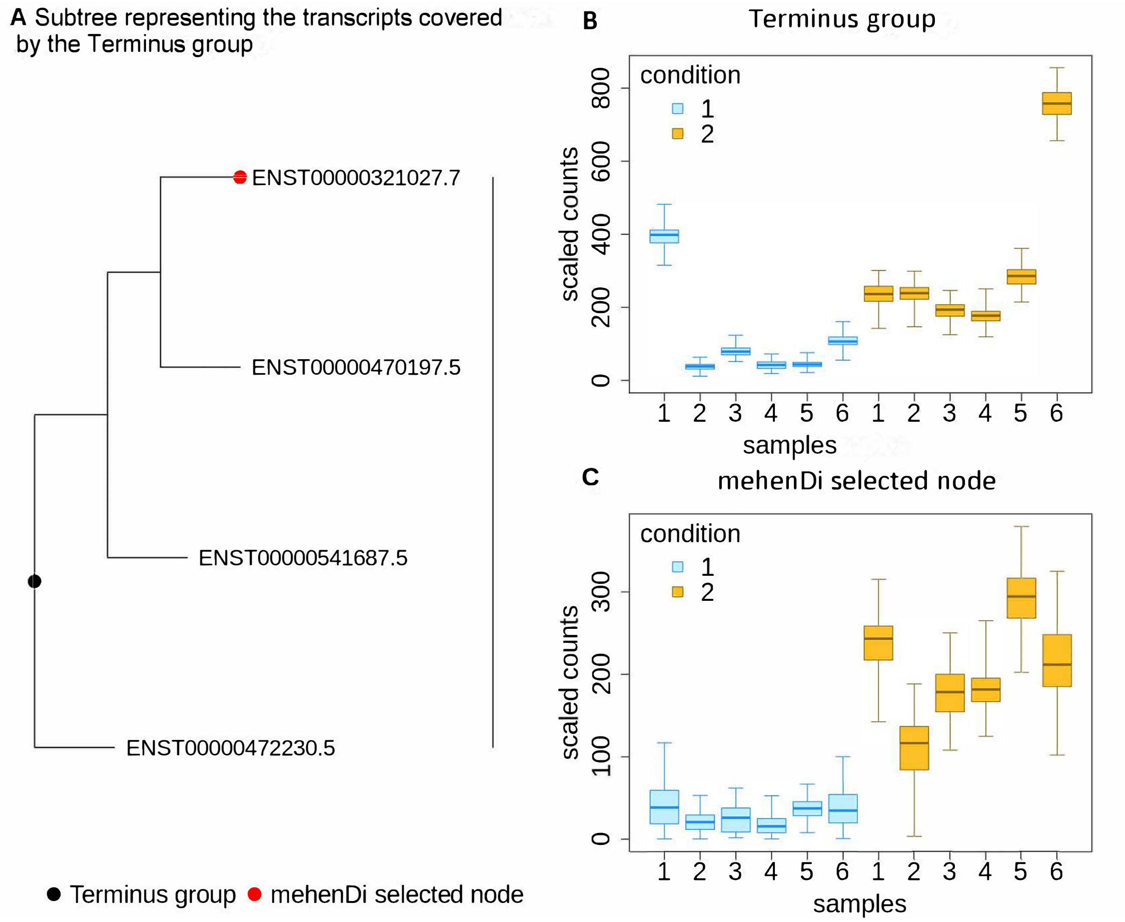

Figure S7: Example of a `mehenDi` node that is not aggregated enough in `Terminus` for the `BrSimNorm` dataset. (A) Subtree representing the transcripts covered by the `mehenDi` group. (B) Inferential replicates for the `Terminus` group. (C) Inferential replicates for the selected node output by `mehenDi`.

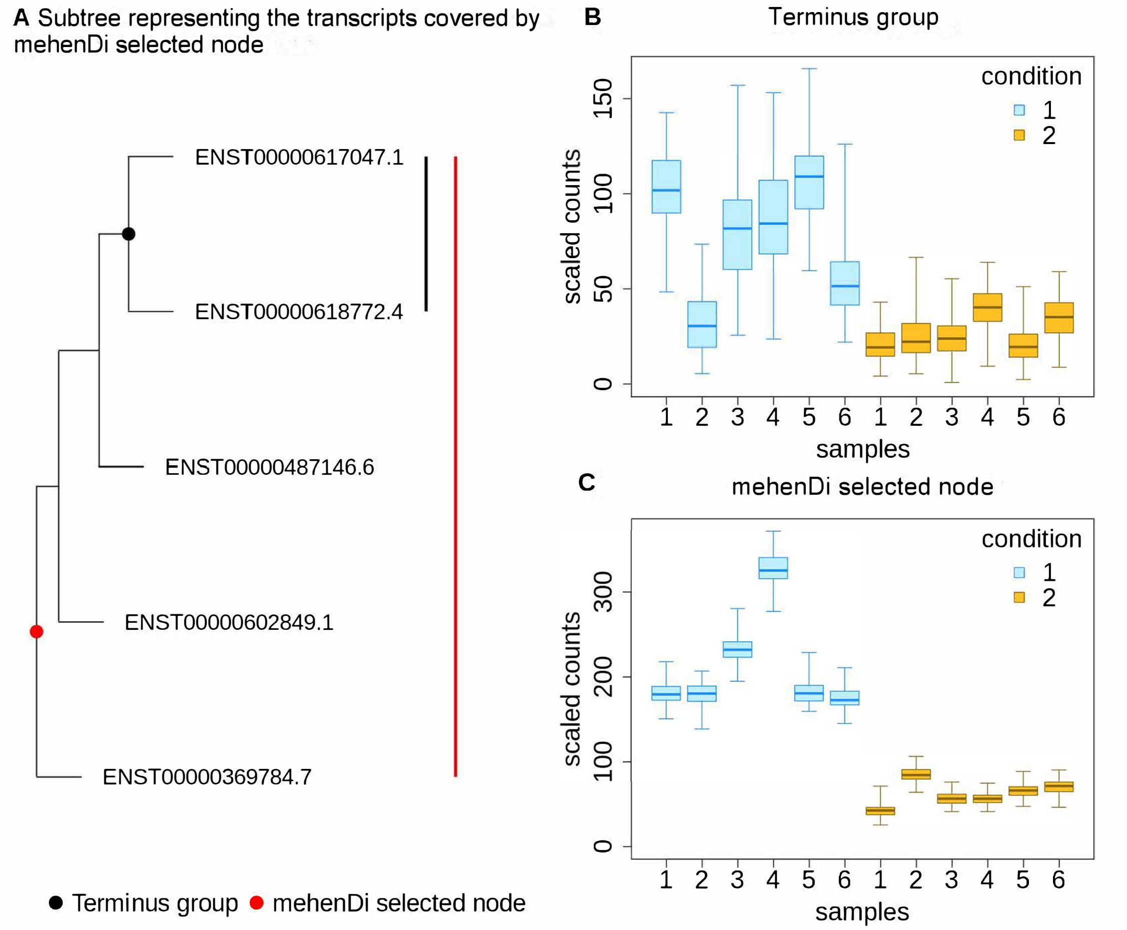

Figure S8: Example of a `mehenDi` node that is not aggregated enough in `Terminus` for the `BrSimNorm` dataset. (A) Subtree representing the transcripts covered by the `mehenDi` group. (B) Inferential replicates for the `Terminus` group. (C) Inferential replicates for the selected node output by `mehenDi`.

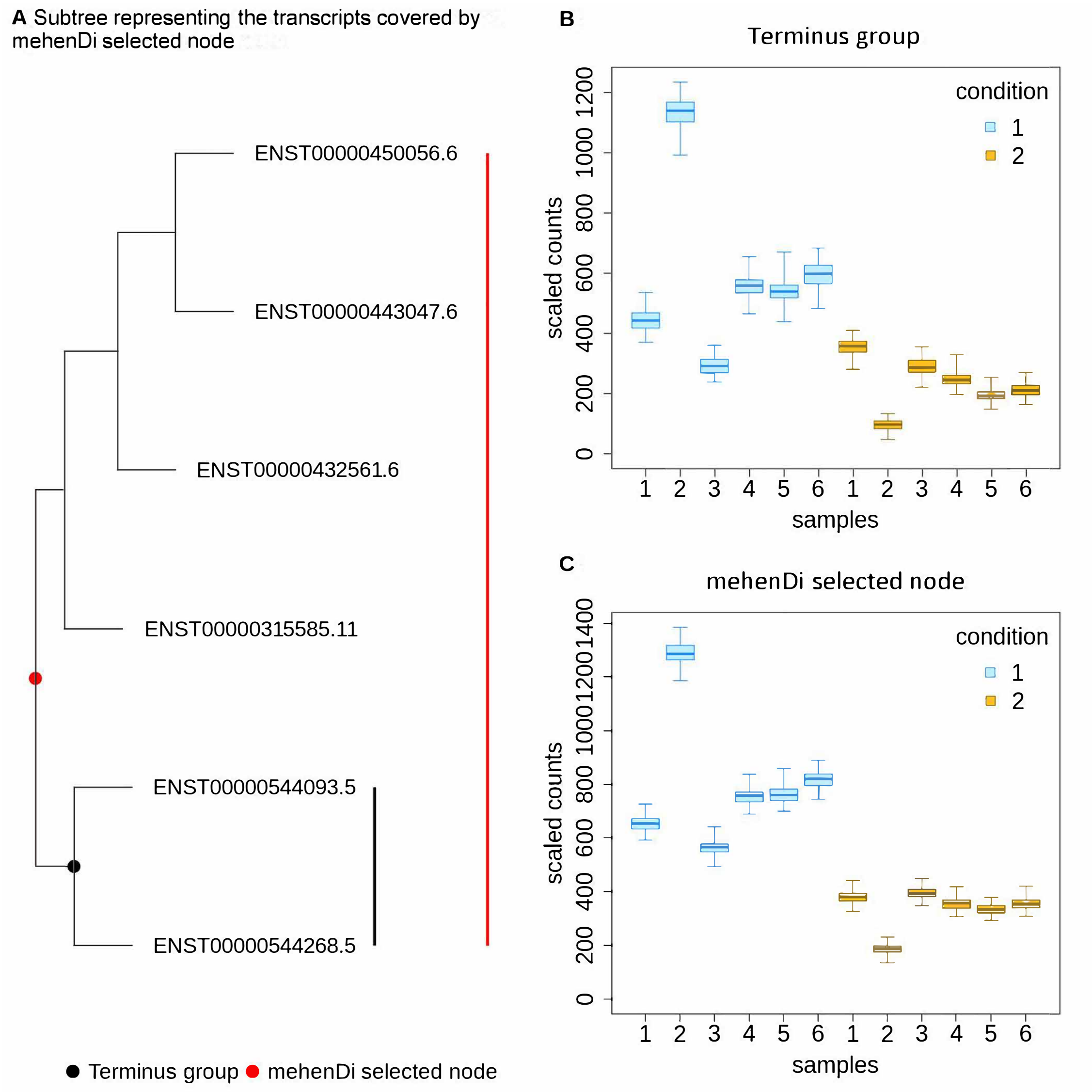

Figure S9: Examination of error metrics for the unique nodes obtained for `treeclimbR` when doing `treeclimbR` vs `Txps` analysis at the different nominal FDR thresholds. We vary the magnitude of log fold change (LFC) and plot the empirical FDR and the total number of nodes that are left after filtering the unique nodes based on LFC.

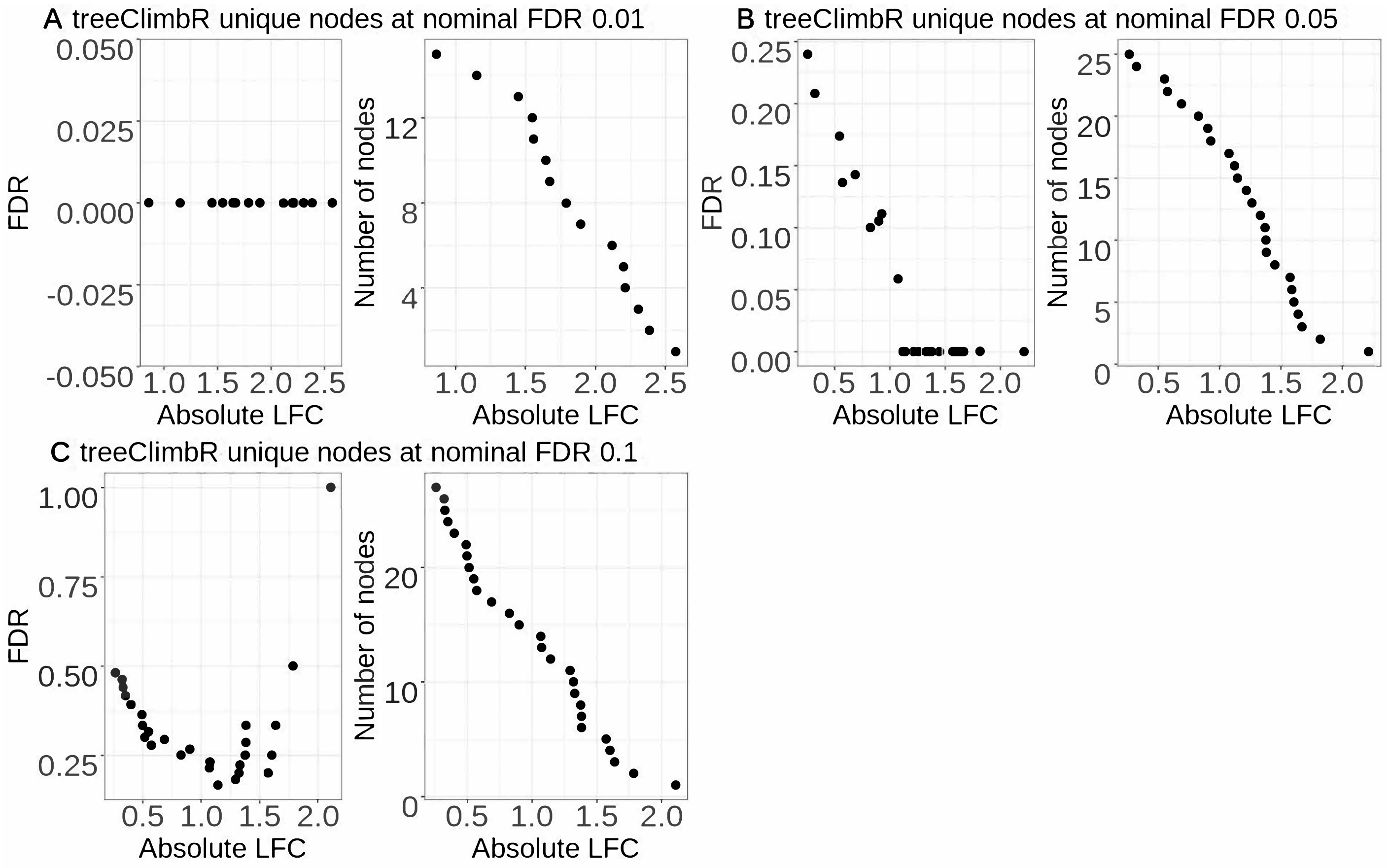

Figure S10: Examination of error metrics for the unique nodes obtained for `Txps` when doing `treeclimbR` vs `Txps` analysis at the different nominal FDR thresholds. We vary the magnitude of log fold change (LFC) and plot the empirical FDR and the total number of nodes that are left after filtering the unique nodes based on LFC.

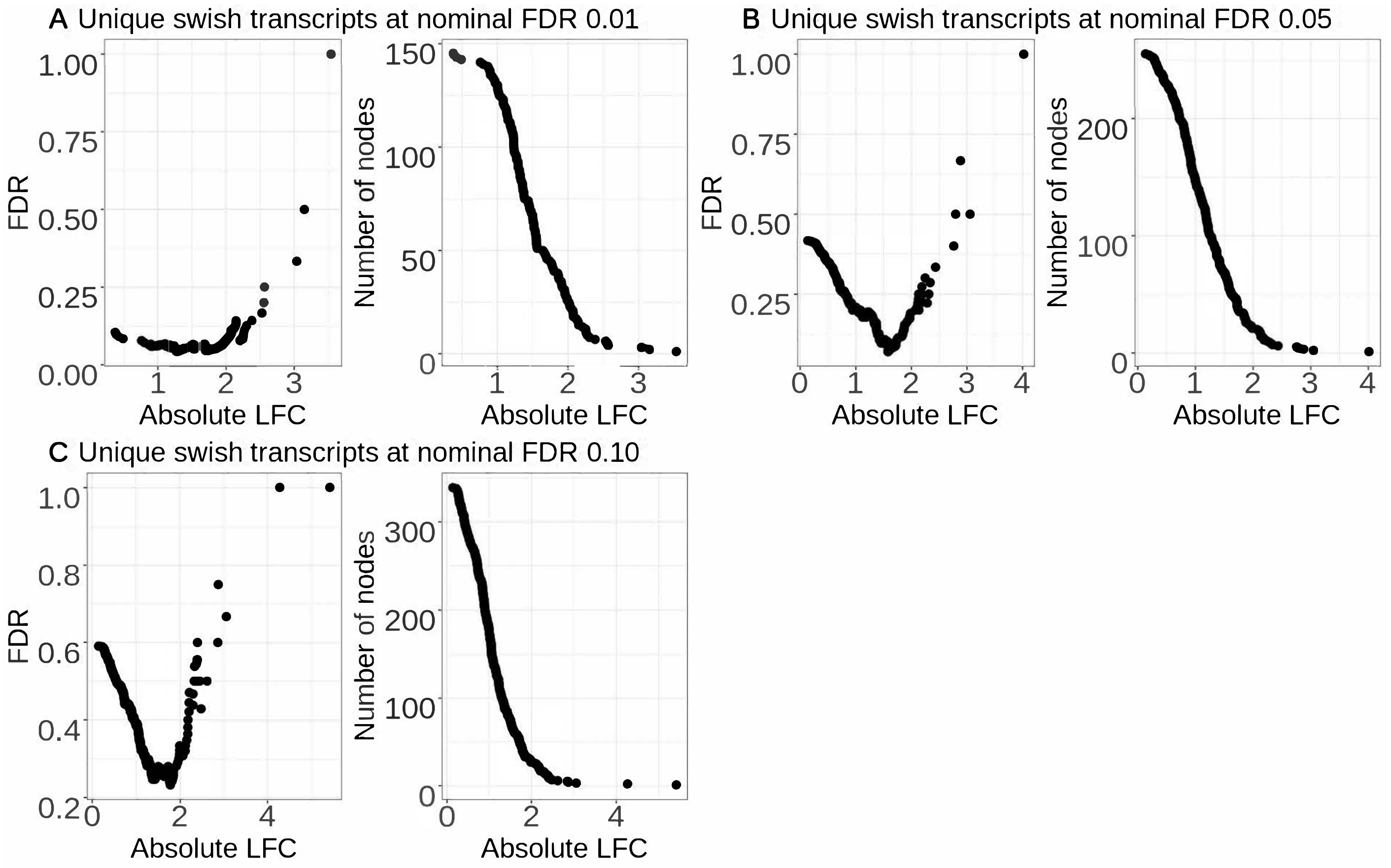

Figure S11: The first two dimensions of the PCA using the top 1000 variable features for the `MouseMuscle` dataset

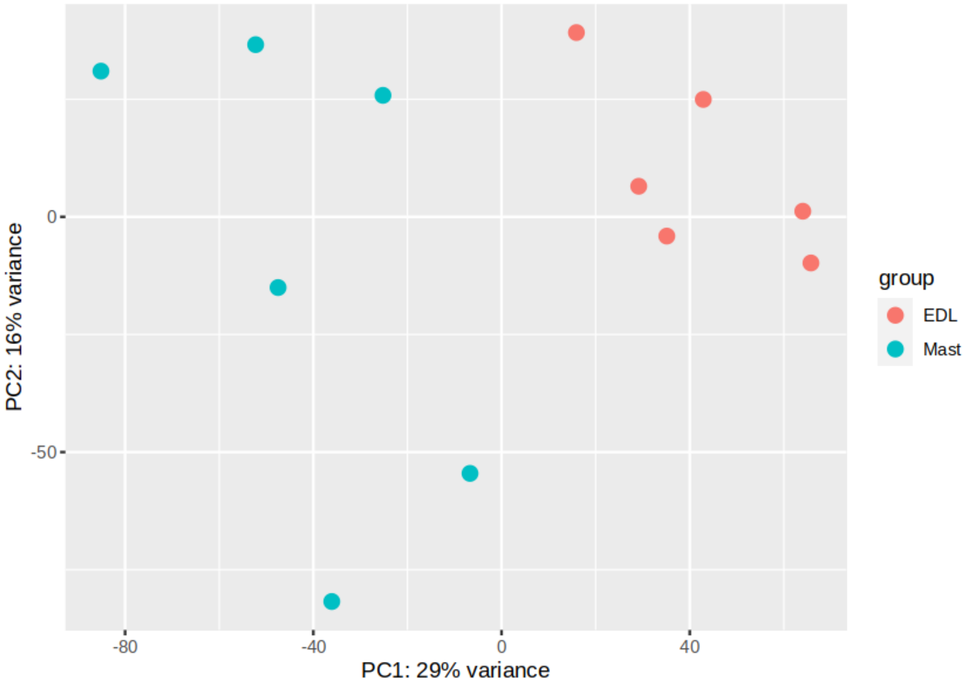

Figure S12: The average distance between the nodes obtained for `mehenDi` using default parameters and varying `minP` and `mIrvThresh` individually for the `MouseMuscle` dataset.

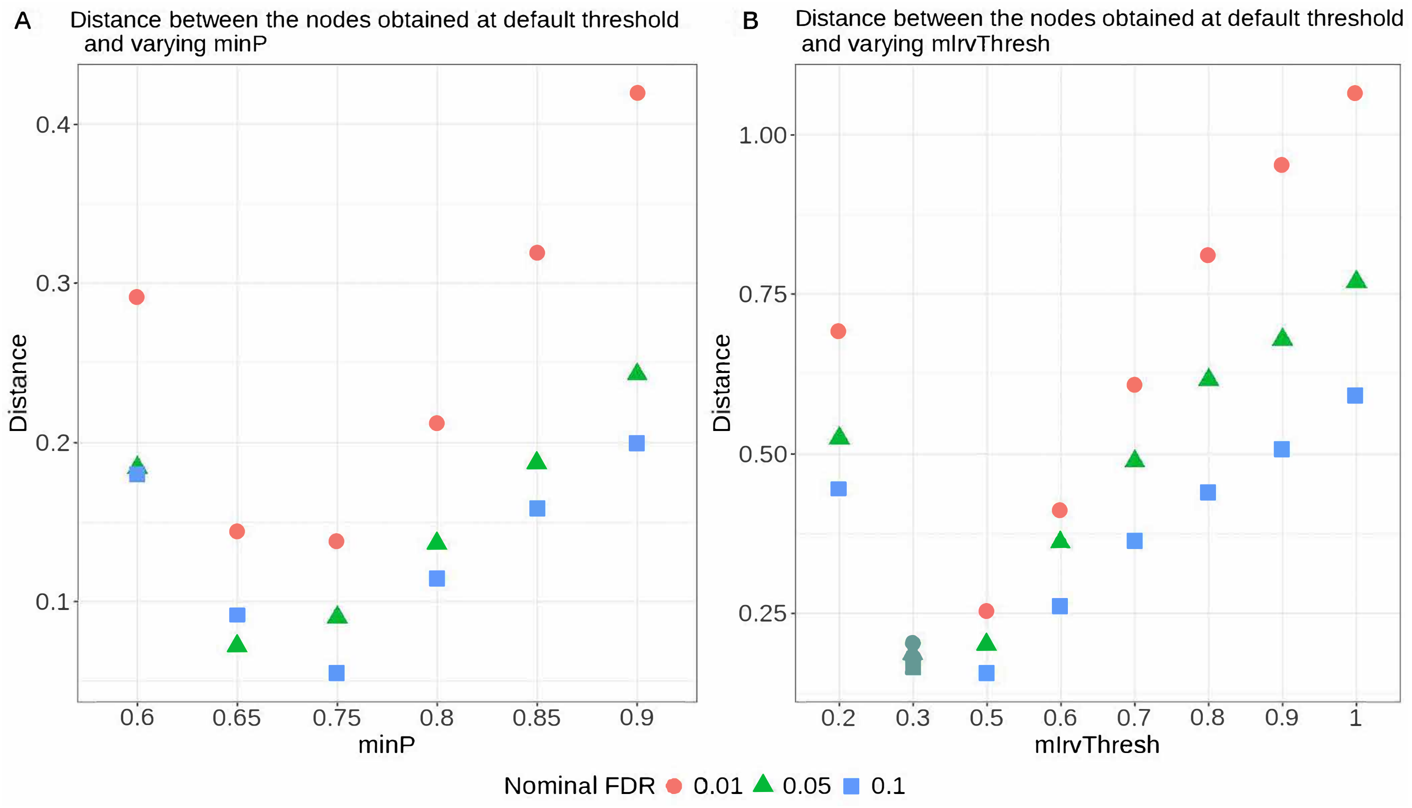

Figure S13: UpSet plot covering the number of transcripts that are covered by the nodes that are output by the different methods for the `MouseMuscle` dataset.

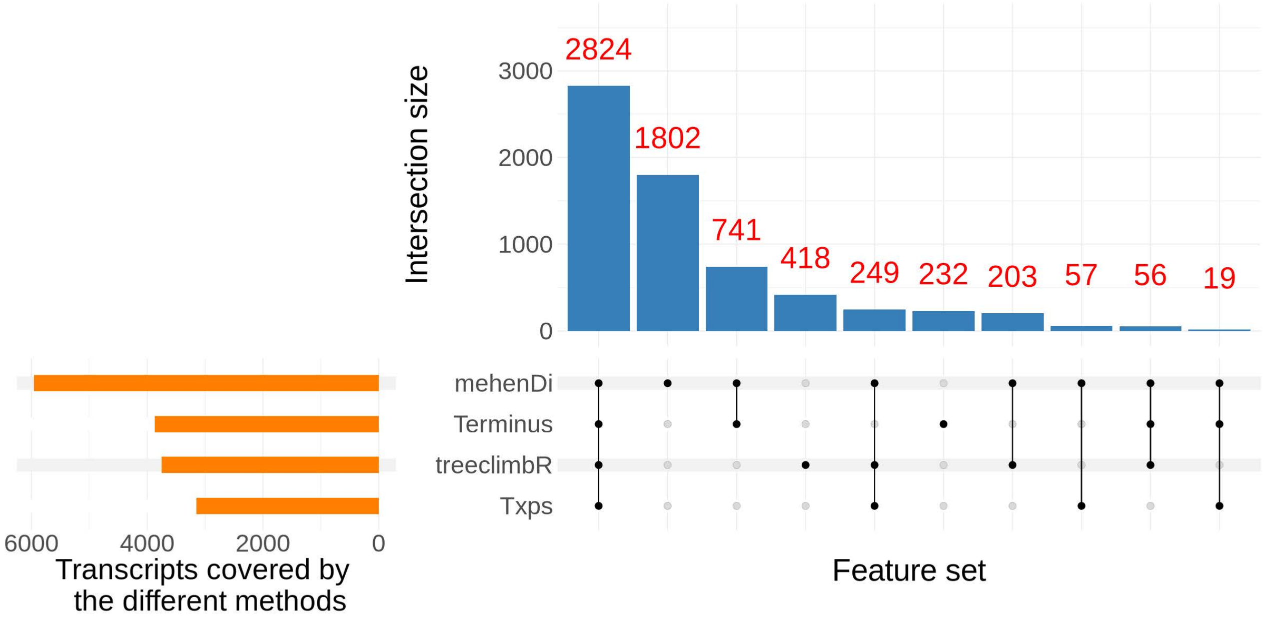

Figure S14: Examining the transcript profile for the gene *Hmcn2* in the `MouseMuscle` dataset. A) Transcripts in a pileup style. B) Tree representing the transcripts covered by the gene *Hmcn2*, with the red node representing the transcripts covered by the `mehenDi` selected node. C) Inferential replicates for the transcript ENSMUST00000138821.7, which had the lowest p-value among all the transcripts in the tree. D) Inferential replicates for the `mehenDi` selected node.

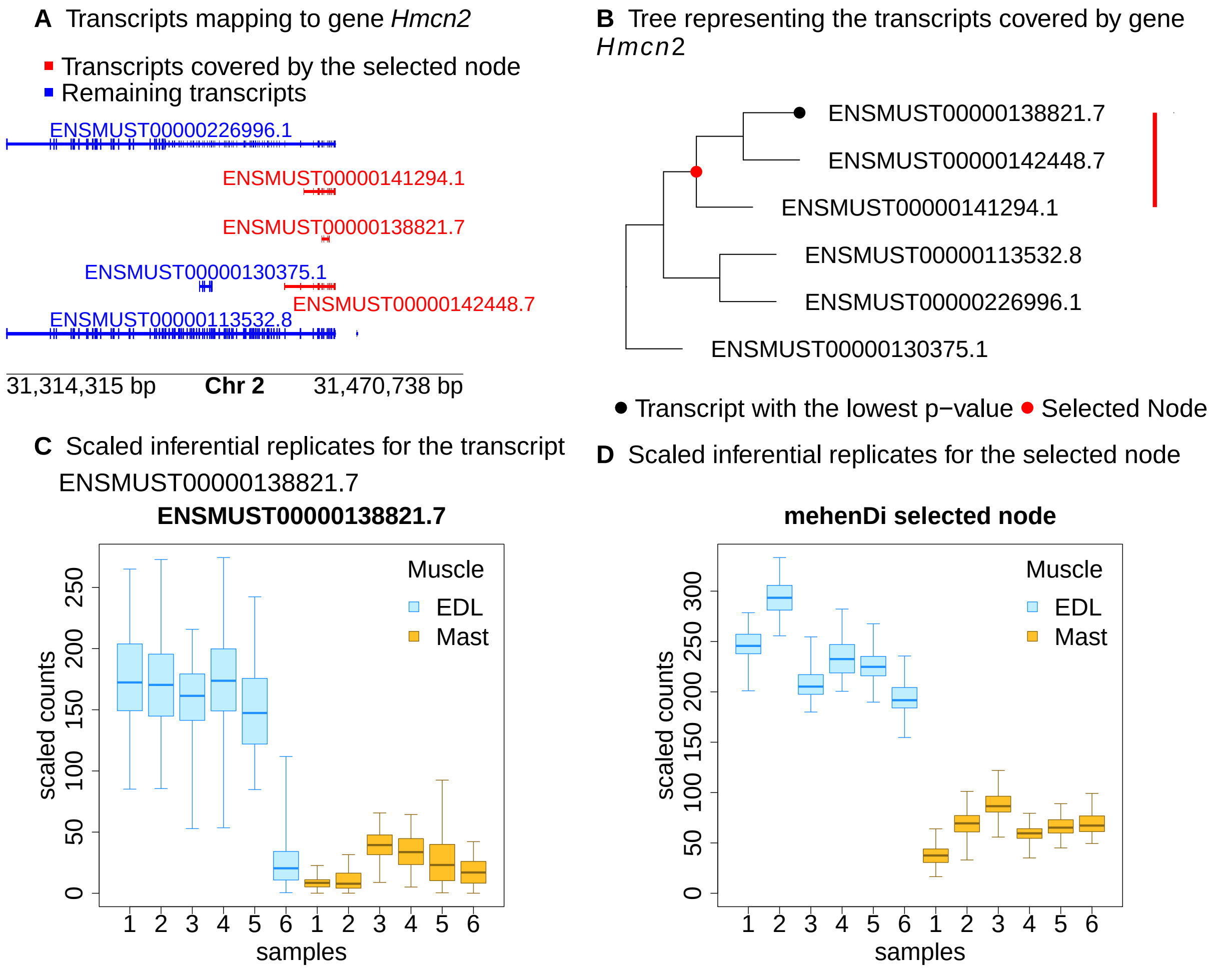

Figure S15: Examining the transcript profile for the gene *Emid1* in the `MouseMuscle` dataset. (A) Transcripts in a pileup style. (B) Tree representing the transcripts covered by the gene *Emid1*, with the red node representing the transcripts covered by the `mehenDi` selected node. (C) Inferential replicates for the transcript ENSMUST00000151906.7, which had the lowest p-value among all the transcripts in the tree. (D) Inferential replicates for the `mehenDi` selected node.

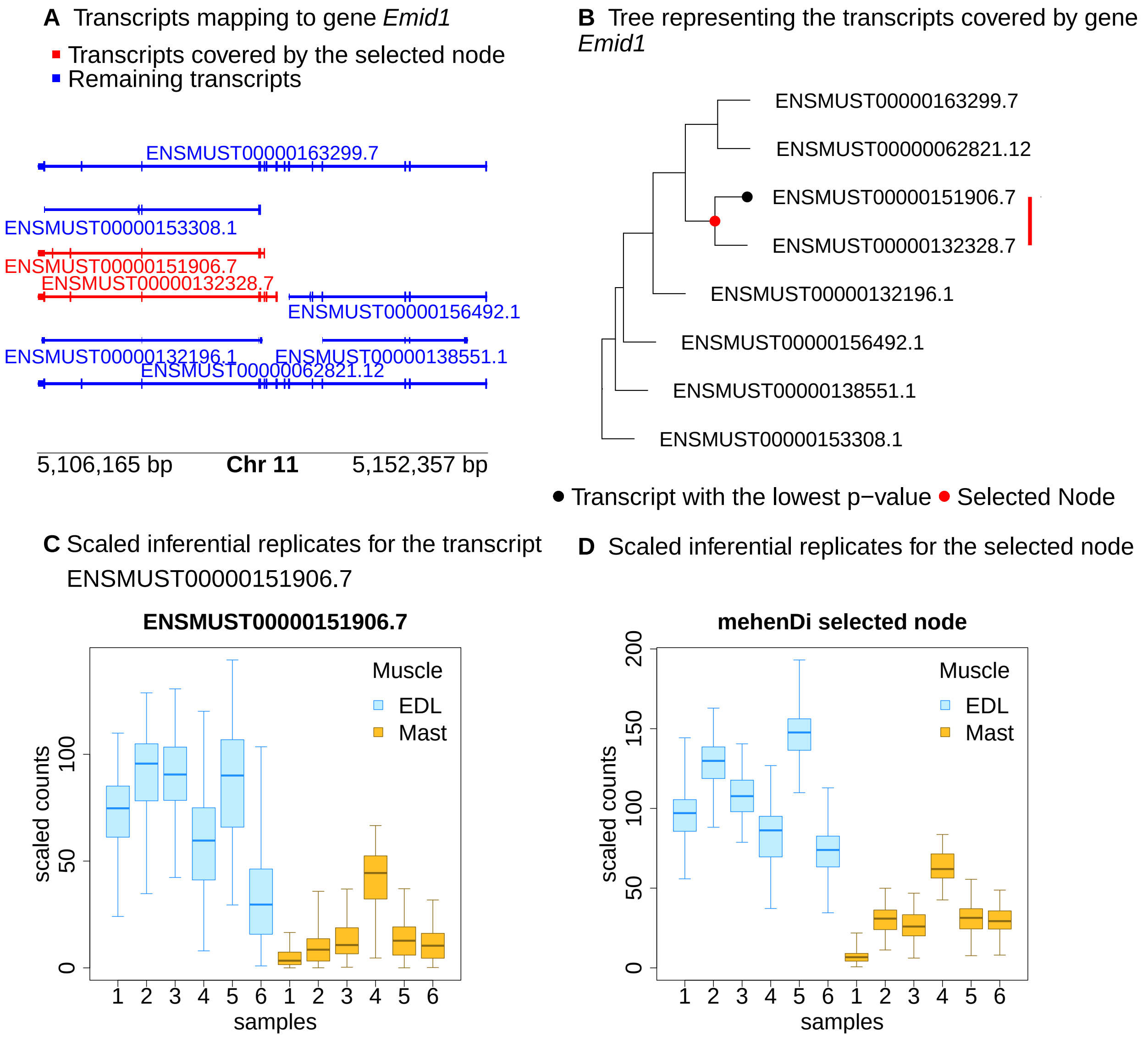

Figure S16: Examining the transcript profile for the gene *Prss55* in the `MouseMuscle` dataset. (A) Transcripts in a pileup style. (B) Tree representing the transcripts covered by the gene *Prss55*, with the red node representing the transcripts covered by the `mehenDi` selected node. (C) Inferential replicates for the transcript ENSMUST00000171503.7, which had the lowest p-value among all the transcripts in the tree. (D) Inferential replicates for the `mehenDi` selected node.

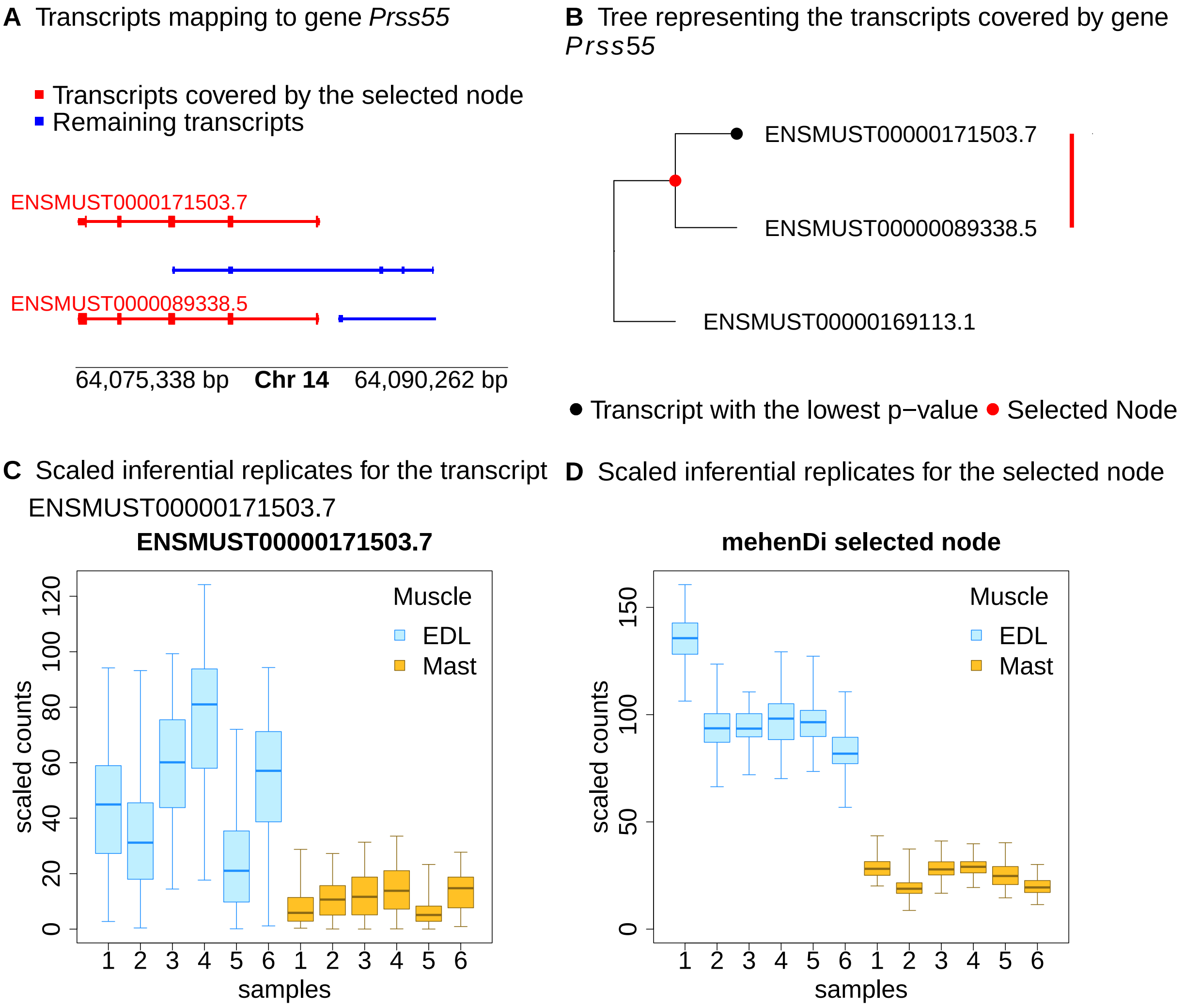

Figure S17: Examining the transcript profile for the gene *Stard10* in the `MouseMuscle` dataset. (A) Transcripts in a pileup style. (B) Tree representing the transcripts covered by the gene *Stard10*, with the red node representing the transcripts covered by the `mehenDi` selected node. (C) Inferential replicates for the transcript ENSMUST000000032927.13, which had the lowest p-value among all the transcripts in the tree. (D) Inferential replicates for the `mehenDi` selected node.

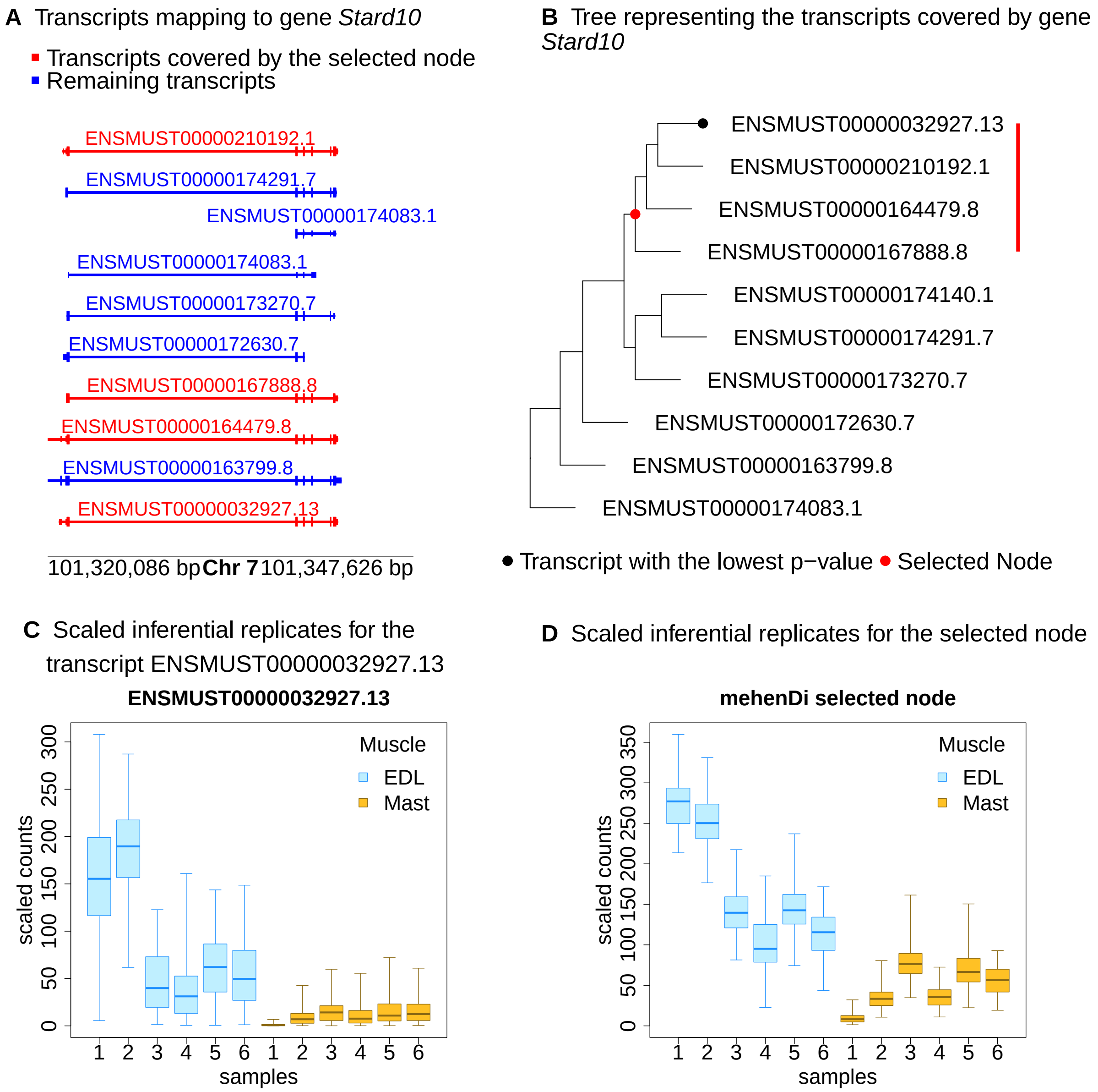

Figure S18: Examining the transcript profile for the gene *Syk* in the `MouseMuscle` dataset. (A) Transcripts in a pileup style. (B) Tree representing the transcripts covered by the gene *SYK*, with the red node representing the transcripts covered by the `mehenDi` selected node. (C) Inferential replicates for the transcript ENSMUST00000055087.6, which had the lowest p-value among all the transcripts in the tree. (D) Inferential replicates for the `mehenDi` selected node.

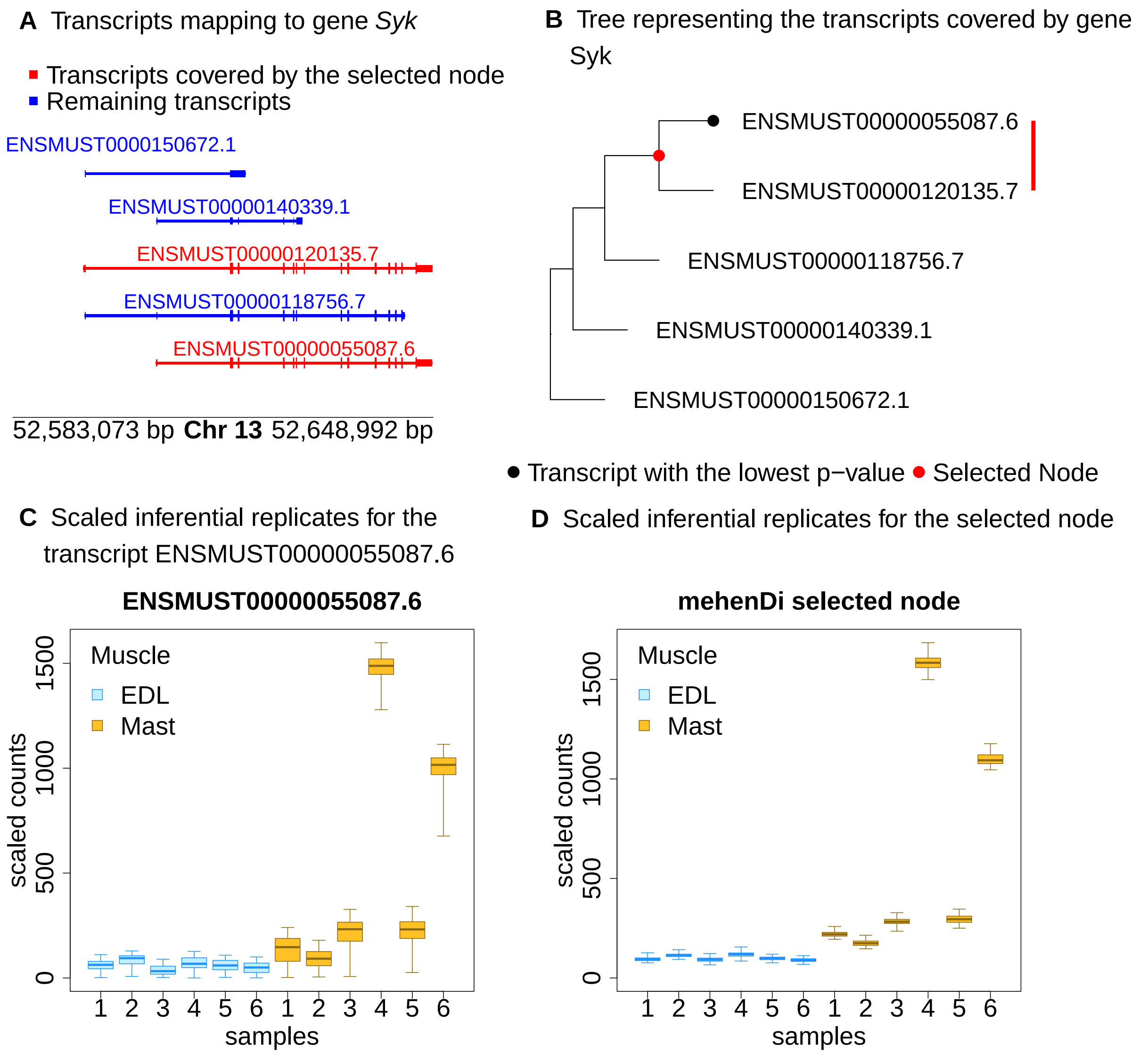

Figure S19: UpSet plot covering the number of transcripts that are covered by the nodes that are output by the different methods for the `ChimpBrain` dataset.

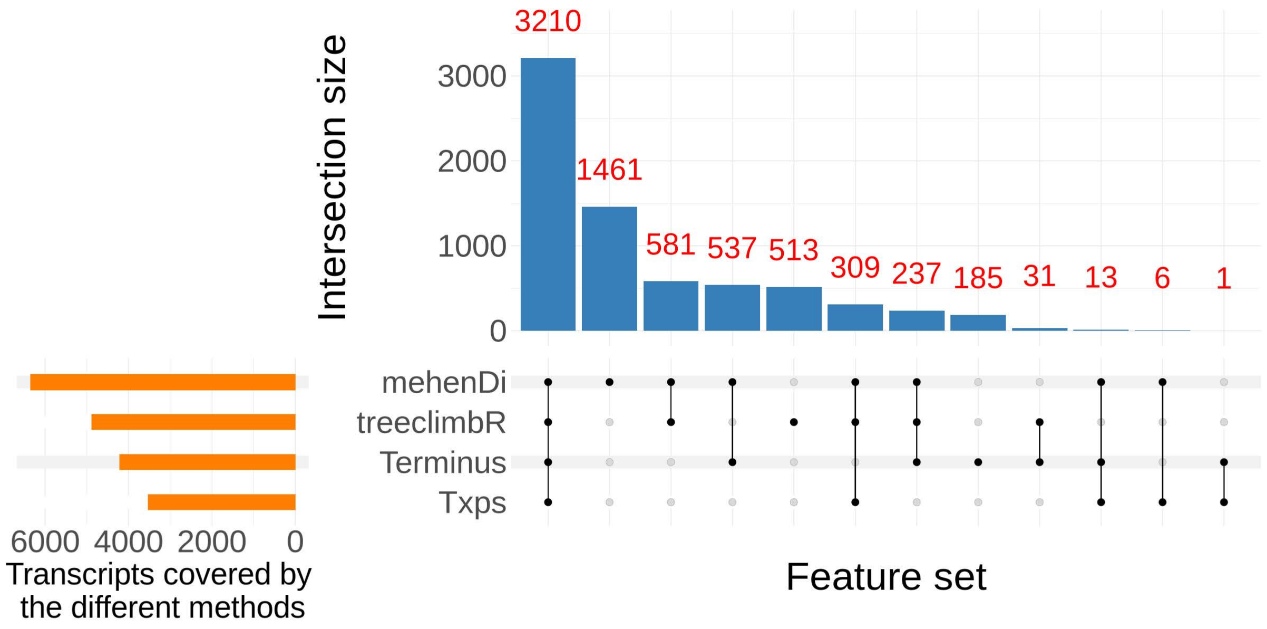

Figure S20: Examining the transcript profile for the gene *CABIN1* in the `ChimpBrain` dataset. (A) Transcripts in a pileup style. (B) Tree representing the transcripts covered by the gene *CABIN1*, with the red node representing the transcripts covered by the `mehenDi` selected node. (C) Inferential replicates for the transcript ENSMUST00000103768, which had the lowest p-value among all the transcripts in the tree. (D) Inferential replicates for the `mehenDi` selected node.

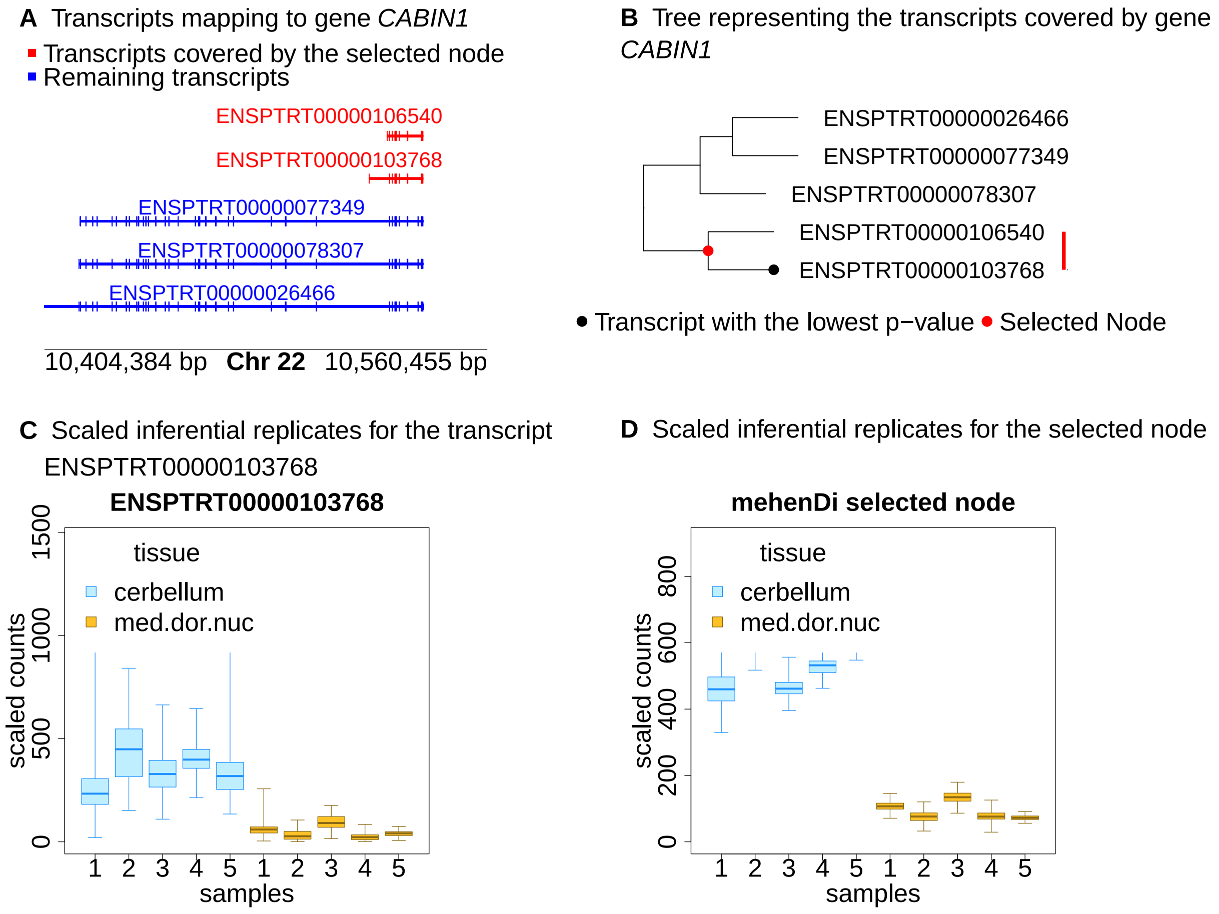

Figure S21: Examining the transcript profile for the gene *CATSPERG* in the `ChimpBrain` dataset. (A) Transcripts in a pileup style. (B) Tree representing the transcripts covered by the gene *CATSPERG*, with the red node representing the transcripts covered by the `mehenDi` selected node. (C) Inferential replicates for the transcript ENSMUST00000020240, which had the lowest p-value among all the transcripts in the tree. (D) Inferential replicates for the `mehenDi` selected node.

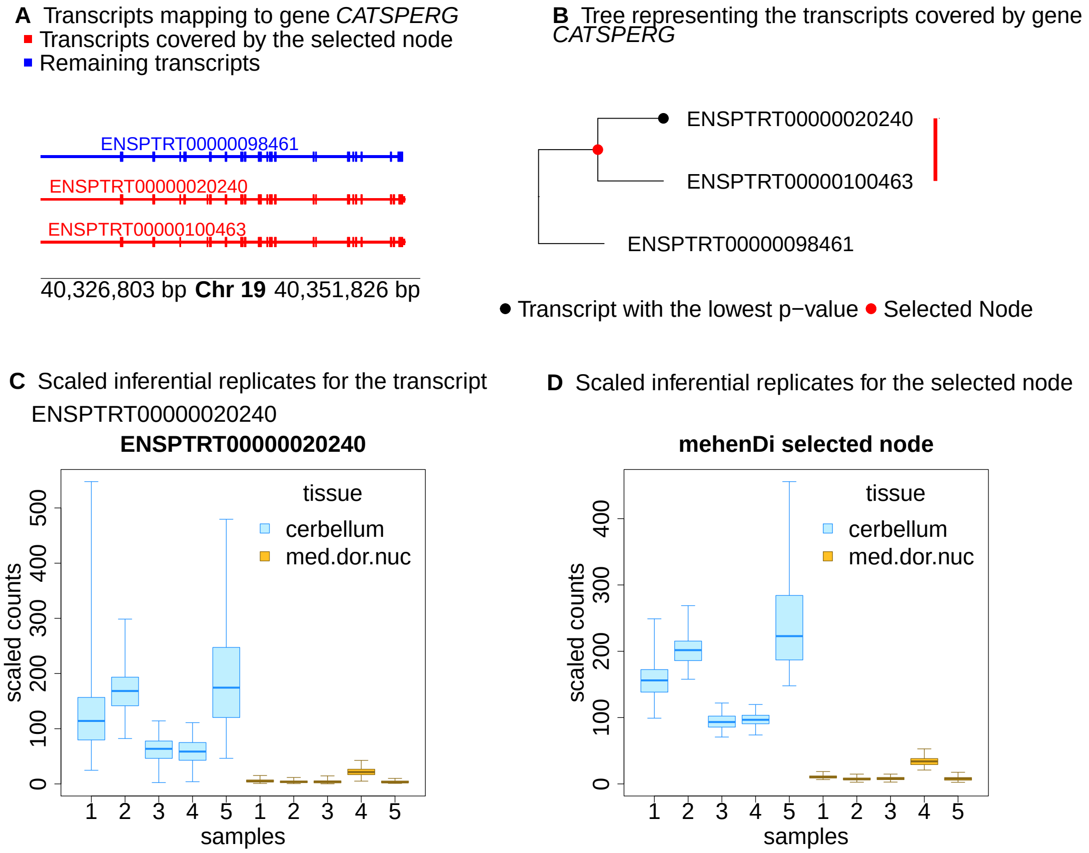

Figure S22: Examining the transcript profile for the gene *EYA1* in the `ChimpBrain` dataset. (A) Transcripts in a pileup style. (B) Tree representing the transcripts covered by the gene *EYA1*, with the red node representing the transcripts covered by the `mehenDi` selected node. (C) Inferential replicates for the transcript ENSMUST00000097751, which had the lowest p-value among all the transcripts in the tree. (D) Inferential replicates for the `mehenDi` selected node.

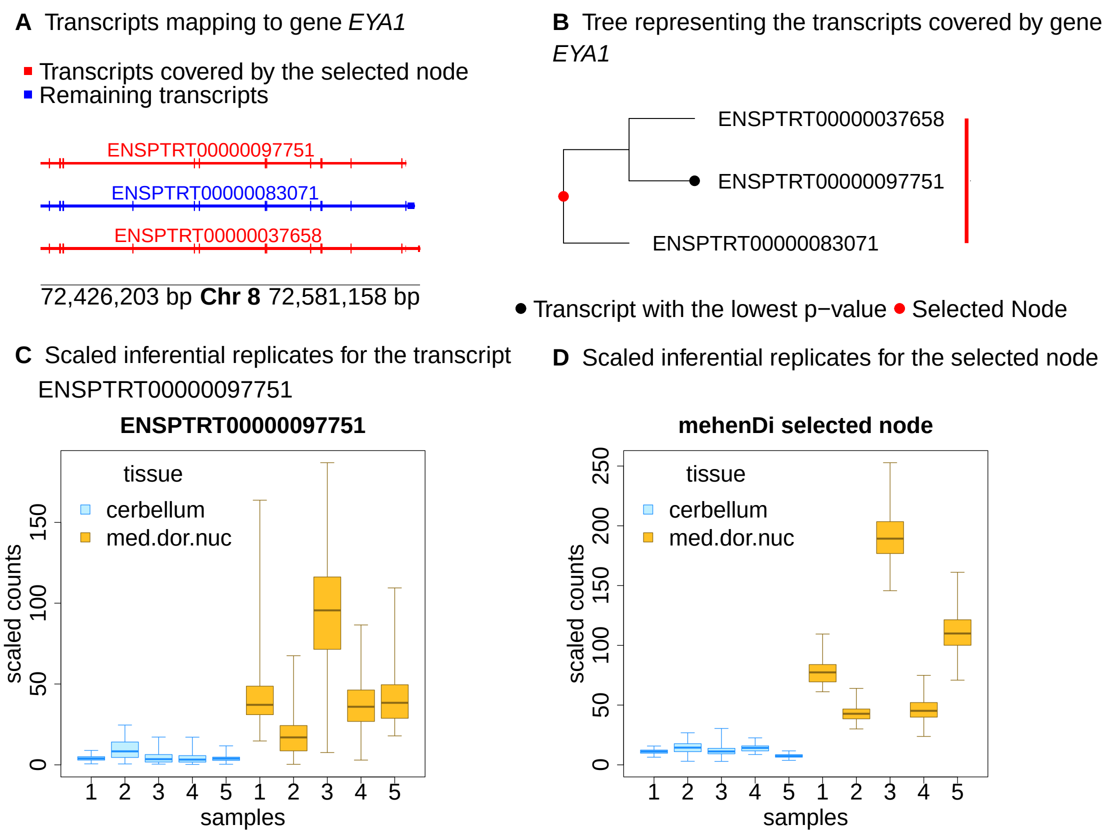

Figure S23: Examining the transcript profile for the gene *MCF2* in the `ChimpBrain` dataset. (A) Transcripts in a pileup style. (B) Tree representing the transcripts covered by the gene *MCF2*, with the red node representing the transcripts covered by the `mehenDi` selected node. (C) Inferential replicates for the transcript ENSMUST00000105901, which had the lowest p-value among all the transcripts in the tree. (D) Inferential replicates for the `mehenDi` selected node.

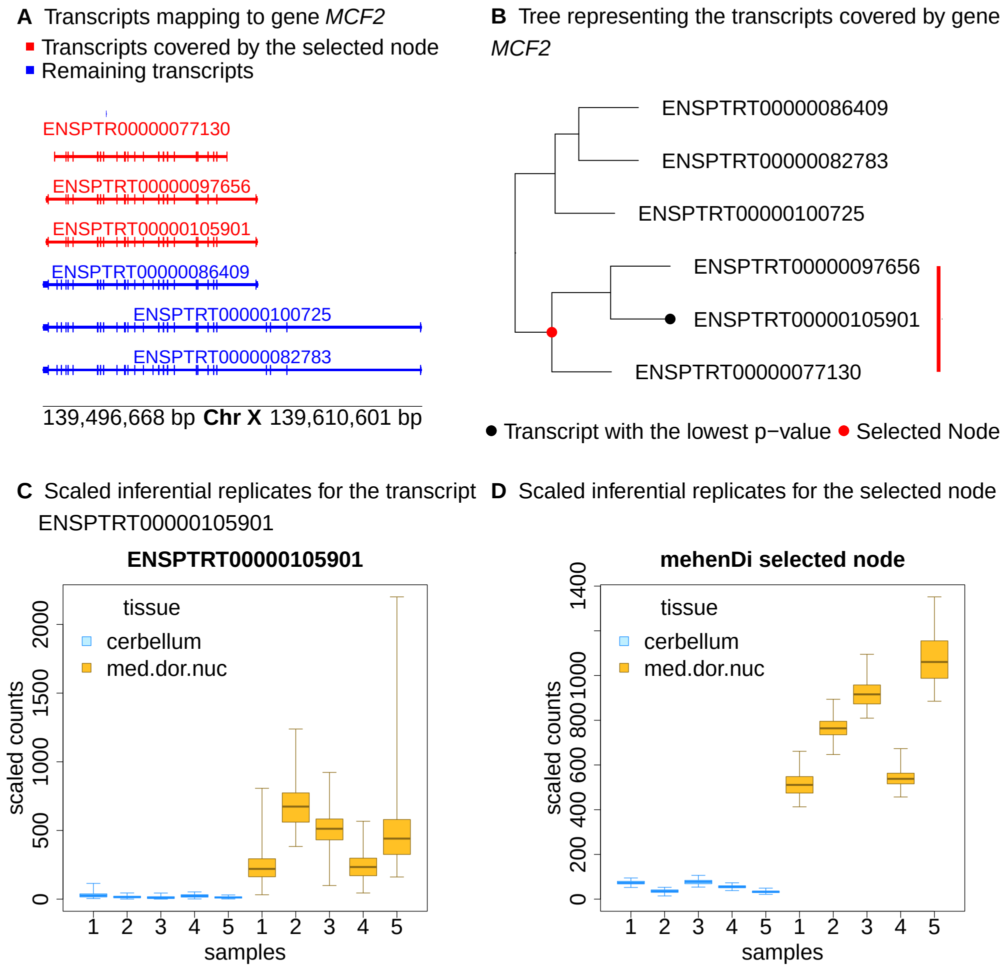

Figure S24: Examining the transcript profile for the gene *MYO5C* in the `ChimpBrain` dataset. (A) Transcripts in a pileup style. (B) Tree representing the transcripts covered by the gene *MYO5C*, with the red node representing the transcripts covered by the `mehenDi` selected node. (C) Inferential replicates for the transcript ENSMUST00000013074, which had the lowest p-value among all the transcripts in the tree. (D) Inferential replicates for the `mehenDi` selected node.

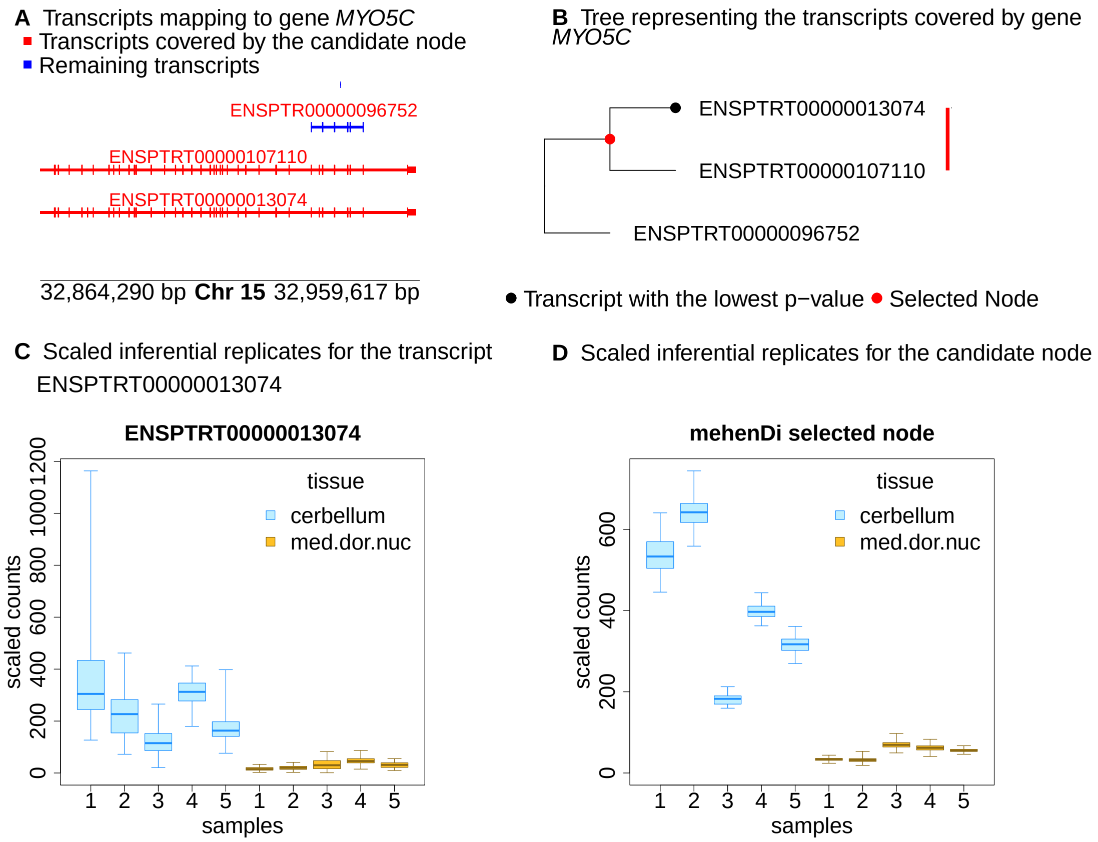

Figure S25: Examining the transcript profile for the gene *PIEZO2* in the `ChimpBrain` dataset. (A) Transcripts in a pileup style. (B) Tree representing the transcripts covered by the gene *PIEZO2*, with the red node representing the transcripts covered by the `mehenDi` selected node. (C) Inferential replicates for the transcript ENSMUST00000094046, which had the lowest p-value among all the transcripts in the tree. (D) Inferential replicates for the `mehenDi` selected node.

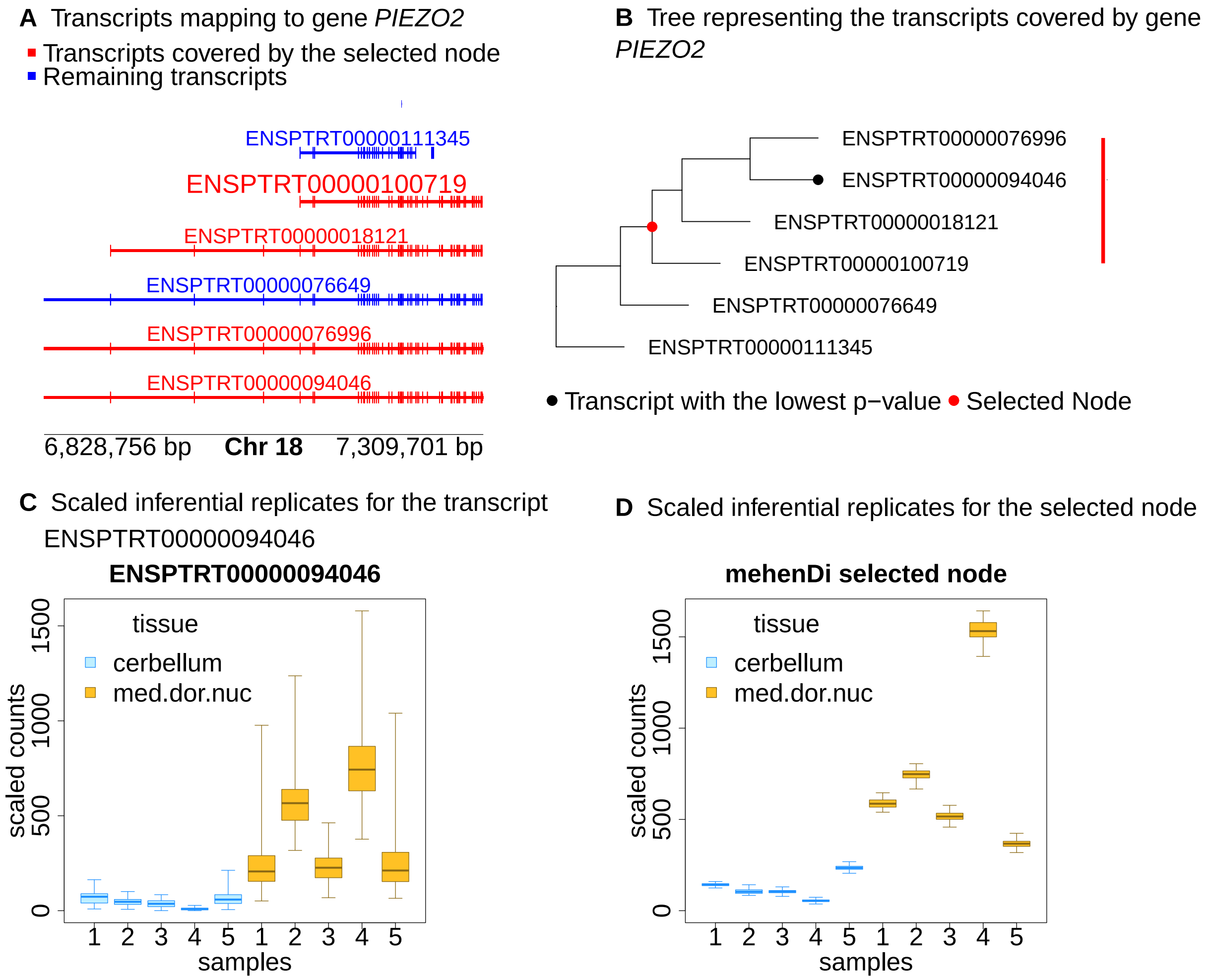

Supplement: Supplement 3 [file Supplemental_Material.pdf]
